# Current trends and future directions in internalized weight stigma research: a scoping review and synthesis of the literature

**DOI:** 10.1186/s40337-024-01058-0

**Published:** 2024-07-15

**Authors:** Sarah Nutter, Jessica F. Saunders, Rachel Waugh

**Affiliations:** 1 Educational Psychology and Leadership Studies, University of Victoria, 3800 Finnerty Road, Victoria, BC V8P 5C2 Canada; 2 Institute on Aging and Lifelong Health, University of Victoria, 3800 Finnerty Road, Victoria, BC V8P 5C2 Canada; 3 Psychology Convening Group, Ramapo College of New Jersey, 505 Ramapo Valley Road, Mahwah, NJ 07430 USA

**Keywords:** Internalized weight stigma, Weight bias internalization, Internalized weight bias, Weight stigma, Scoping review

## Abstract

**Background:**

Since the first papers focused on internalized weight stigma were published in the mid 2000’s, the literature has grown into a robust field that complements existing knowledge on weight stigma. Recently, researchers have documented the need for increased conceptual and measurement clarity, to distinguish internalized weight stigma from body dissatisfaction. Although several systematic reviews have been conducted on portions of the internalized weight stigma literature, no review to date has been conducted examining the entirety of the literature.

**Objective:**

The aim of this research was to conduct a systematic scoping review and synthesis of research on internalized weight stigma. Specifically, we sought to examine the broad scope of the literature, terms used to refer to internalized weight stigma, how internalized weight stigma is defined, sample characteristics, and weight-based framings of internalized weight stigma research.

**Methods:**

We conducted a single-concept search across six databases (EMBASE, Medline, PsychINFO, PubMed, SCOPUS, and Web of Science) of peer-reviewed papers published in English on internalized weight stigma. Data were extracted for article authors, year published, journal name and type, general article topic(s), study design, study location, sample characteristics, variables measured, paper framing, term used to describe internalized weight stigma, and definition of internalized weight stigma.

**Results:**

Of the 931 unique records screened, 376 were identified for inclusion in the scoping review. The majority of internalized weight stigma research is characterized by cross-sectional methods, has been conducted in the US, and has utilized samples of higher weight white women. Further, 40 unique terms were used across the literature to refer to internalized weight stigma, and 19 different components of definitions of internalized weight stigma were identified. The literature is also characterized by a focus on understanding the association between internalized weight stigma and health outcomes with an emphasis on obesity.

**Conclusions:**

This scoping review confirms a lack of concept clarity of internalized weight stigma, in part influenced by an inconsistency in definitions of internalized weight stigma across the literature. Considerations are provided for steps to enhance conceptual and measurement clarity. Given the obesity focused framing of much of the research on internalized weight stigma, considerations are also provided for reducing weight-centric approaches to research.

**Plain english summary:**

In the early 2000’s, researchers began to pay more attention to the potential health impacts of believing societal stereotypes, negative attitudes, and beliefs about higher weight people. When these stereotypes, negative attitudes, and beliefs are directed towards the self, it can have significant consequences for an individual’s perceptions of self. This research collected and summarized all existing research published in English on internalized weight stigma. Our results highlighted that researchers do not use consistent terminology to refer to internalized weight stigma and that they do not have a consistent definition of internalized weight stigma. Further, a large proportion of the research is focused on obesity or weight loss, which may unintentionally perpetuate weight stigma in scientific research. We provide several recommendations for researchers to address these challenges in future research on internalized weight stigma as well as recommendations to address other identified gaps in the existing literature.

**Supplementary Information:**

The online version contains supplementary material available at 10.1186/s40337-024-01058-0.

## Introduction

Weight stigma is defined as encompassing the stereotypes as well as the negative beliefs and judgements towards higher weight people [1]. Weight stigma literature is a large and rich body of research that has grown exponentially in the last few decades, such that the last comprehensive review of the entire literature was published in 2009 [2]. Subsequent reviews have focused on subsets of the literature such as the impact of weight stigma on health [3], the extent and impact of weight stigma in educational settings [4] and among healthcare professionals [5], the psychometric properties of scales measuring weight stigma [6], and the exploration of policy implementation and support to reduce weight stigma [7]. Weight stigma has been identified as an important social justice issue that negatively impacts the equity of higher-weight people [8, 9].

Approximately 20 years ago, researchers recognized that weight-based stereotypes and negative attitudes were commonly held by individuals across the weight spectrum, prompting purposeful exploration of the extent to which higher-weight people hold weight-based stereotypes and judgements, conceptualizing this bias as a self-stigma and internalized attitudes about the self [10–13]. Since then, this phenomenon has been commonly referred to as internalized weight bias, internalized weight stigma, weight bias internalization, and/or weight self-stigma, among other terms [14].

The earliest published papers examining internalized weight stigma used stereotype endorsement as a proxy for the internalization of stereotypes [10–13]. In a sample of 1,081 adults engaged in a commercial weight management program, participants reported what they believed to be the most common existing weight-based stereotypes in society, as well as whether they believed these societal stereotypes to be generally true or not [12]. This method was also used in a qualitative study examining experiences with weight stigma among 318 adults engaged in a commercial weight management program [13]. Stereotype endorsement was also examined in a longitudinal study with 163 adolescent girls at ages 9 and 11 years [11], as well as in a sample of 58 higher-weight adults [10]. In these studies, internalized weight stigma was operationalized as stereotype endorsement among higher-weight participants. In this early research, endorsement of weight stereotypes was examined in association with health-related outcomes, such as coping strategies, emotional well-being, self-worth, binge eating, and eating attitudes [10–12].

These early publications were followed by the development of the Weight Bias Internalization Scale (WBIS) [15] and the Weight Self-Stigma Questionnaire (WSSQ) [16]. In developing both scales, researchers recognized that existing measures of explicit weight stigma directed towards others may not accurately or adequately capture internalized weight stigma when administered to higher-weight participants, as individuals may hold stereotypes and negative attitudes about others, but not themselves. The WBIS is a 11-item measure, developed to examine internalized weight stigma among higher-weight individuals [15], and it has since been modified for use with individuals across the weight spectrum [17]. The WSSQ is a 12-item measure also designed for use with higher-weight participants across two factors: self-devaluation and fear of enacted stigma [16]. Both scales have since been translated into several languages, including Traditional Chinese (Taiwan) [18], Spanish [19], and Japanese [20], as well as adapted for use with adolescents [21], children [22], and pregnant participants [23].

Since the introduction of the WBIS [15], its modification (WBIS-M; [24]), and the development of the WSSQ [16], the literature on internalized weight stigma has developed into a robust field, complementing the broader knowledge base on weight stigma. Research on internalized weight stigma has provided a better understanding of the impact of weight stigma on individuals across the weight spectrum. Much of this research has focused on health-related correlates and outcomes associated with internalized weight stigma, including depression, anxiety, self-esteem, body image, eating behaviours and pathology, health-related quality of life, and physical activity [17]. Previous systematic reviews have been conducted to synthesize internalized weight stigma research in relation to its specific impacts such as on health [17], including biopsychosocial outcomes [25, 26], psychological distress [26, 27], health behaviours [26, 28], and mental health in youth [29]. Other systematic reviews have examined the impact of acceptance and commitment therapy on internalized weight stigma [30], as well as the changes in internalized or experienced weight stigma after bariatric surgery [31]. Despite these previous systematic reviews and meta-analyses, no scoping review has been conducted on the entirety of the literature on internalized weight stigma. Scoping reviews provide a useful opportunity to synthesize the size and nature of a body of knowledge and can aid researchers in identifying research gaps and priorities for the future [32]. Without such a review, it is difficult to know how much research has been published on internalized weight stigma, the topic areas that have been explored in the literature, the methodological approaches used, and what populations are currently represented in extant literature.

## Current study

Recently, researchers have examined the measurement overlap between internalized weight stigma and body dissatisfaction, prompting calls for further examination into whether these constructs are independent, or if they represent a jangle fallacy (i.e., when the same construct has been similarly conceptualized in multiple ways [14, 33–35]). In one recent study, internalized weight stigma and body image were found to uniquely contribute to psychological outcomes of self-esteem, depression, and body shame among a sample of racially diverse participants [36]. Overall, these researchers have noted that greater conceptual and measurement clarity into internalized weight stigma, as a construct distinct from body dissatisfaction, is needed [14, 33–36]. A significant factor in the conceptualization and measurement of internalized weight stigma may be the inconsistency in the terms used to refer to internalized weight stigma, as well as varying definitions across the literature [14]. Thus, a scoping review will help to uncover the scope of this inconsistency and identify the most commonly used terms and definitions.

The purpose of this scoping review was to synthesize the peer-reviewed literature on internalized weight stigma. Specifically, we sought to (1) document the research contexts in which internalized weight stigma is being investigated; (2) better understand the inconsistencies in terminology and definitions used; and (3) identify research gaps and future research directions. Six research questions guided this scoping review:

1. What is the broad scope of internalized weight stigma literature, including year published, study location, as well as methodological approach, and in what peer-reviewed journals is this research being published?
2. What terms are used to refer to internalized weight stigma?
3. How is internalized weight stigma defined by researchers?
4. Who is represented in internalized weight stigma research (i.e., sample characteristics)?
5. What are the general research topics of articles on internalized weight stigma?
6. To what extent is the literature framed in a weight-centric manner?

## Method

This scoping review was guided by methods outlined in the *Cochrane Handbook for Systematic Reviews of Interventions* [37] and used the Preferred Reporting Items for Systematic Reviews and Meta-Analyses scoping review extension checklist (PRISMA-ScR) [32].

### Eligibility criteria

The all-text single-concept search strategy utilized the search terms identified in previous systematic reviews on internalized weight stigma [17, 34]. The following keywords were used, with each term put in quotations within the search: weight bias internalization, weight bias internalisation, weight stigma internalization, weight stigma internalisation, internalized weight bias, internalised weight bias, internalized weight stigma, internalised weight stigma, self-directed weight stigma, self-directed weight bias, weight self-stigma, internalized fat phobia, internalised fat phobia, and self-directed fat phobia. Articles were included in the review if they were peer-reviewed, published in English, and if one of the following criteria were met: (1) internalized weight stigma was quantitatively measured; (2) the study reported on a review (i.e., systematic, scoping, critical, etc.) in which internalized weight stigma was a key concept; (3) qualitative thematic findings related to internalized weight stigma were reported; or (4) the article was a commentary, editorial, or randomized controlled trial protocol in which internalized weight stigma was a key concept or included measure.

### Search strategy and data extraction

An initial search was conducted by the first author (SN) on April 25, 2022, and was updated on January 23, 2023, to include all assigned-to-issue articles published in 2022. A second update was conducted on January 23, 2024, to include all assigned-to-issue articles published in 2023. Articles that were published online ahead-of-print were excluded. Searches were conducted in six databases: EMBASE, Medline, PsychINFO, PubMed, SCOPUS, and Web of Science. All searches were uploaded into Covidence software. Following the removal of duplicates, the first and second authors (SN, JFS) independently screened the title and abstracts of all search results. Conflicts were resolved, as required, by discussion. Full-text review and data extraction was conducted by primarily by the first and second authors (SN, JFS) with support from the third author (RW) on the second updated search. Extracted data included: article authors, year published, journal name, general article topic(s), study design, study location, sample characteristics, variables measured, paper framing (i.e., food addiction, weight loss/control focused, obesity focused), term used to describe internalized weight stigma, and definition of internalized weight stigma used, if any.

## Synthesis of results

Data were extracted into a spreadsheet for synthesis and analysis. Data pertaining to the first research question was analyzed using frequencies. Journal names were extracted and grouped thematically by RW and SN, referring to journal aims and scope when necessary. Research question two was analyzed by extracting the term(s) used to refer to internalized weight stigma by researchers and calculating the frequencies of each term. Most papers used only one term. When more than one term was used, each was extracted and included in the analysis. For research question three, definitions were extracted verbatim from literature reviews or methods sections, if available, and coded for inclusion the following components: (1) internalization of weight-based stereotypes; (2) internalization or acceptance of negative weight-based beliefs or attitudes; (3) self-devaluation; (4) fear of enacted stigma; or (5) other features. Frequency counts for each component of common definitions of internalized weight stigma were then calculated. Data extracted for research question four included participant gender, age, and sexuality, as well as whether the sample was (1) comprised solely of higher-weight individuals; (2) a weight management sample; (3) a surgical sample; (4) or another kind of clinical sample (e.g., individuals with an eating disorder diagnosis). Research question five was analyzed by extracting 1–2 major topics or concepts of focus from article titles and abstracts and were grouped thematically by RW and SN. Finally, research question six was analyzed using frequency counts of ratings conducted by SN and JFS for whether or not articles were framed around the concepts of food addiction, weight loss/control, obesity, or fat acceptance.

## Results

**Fig. 1:**
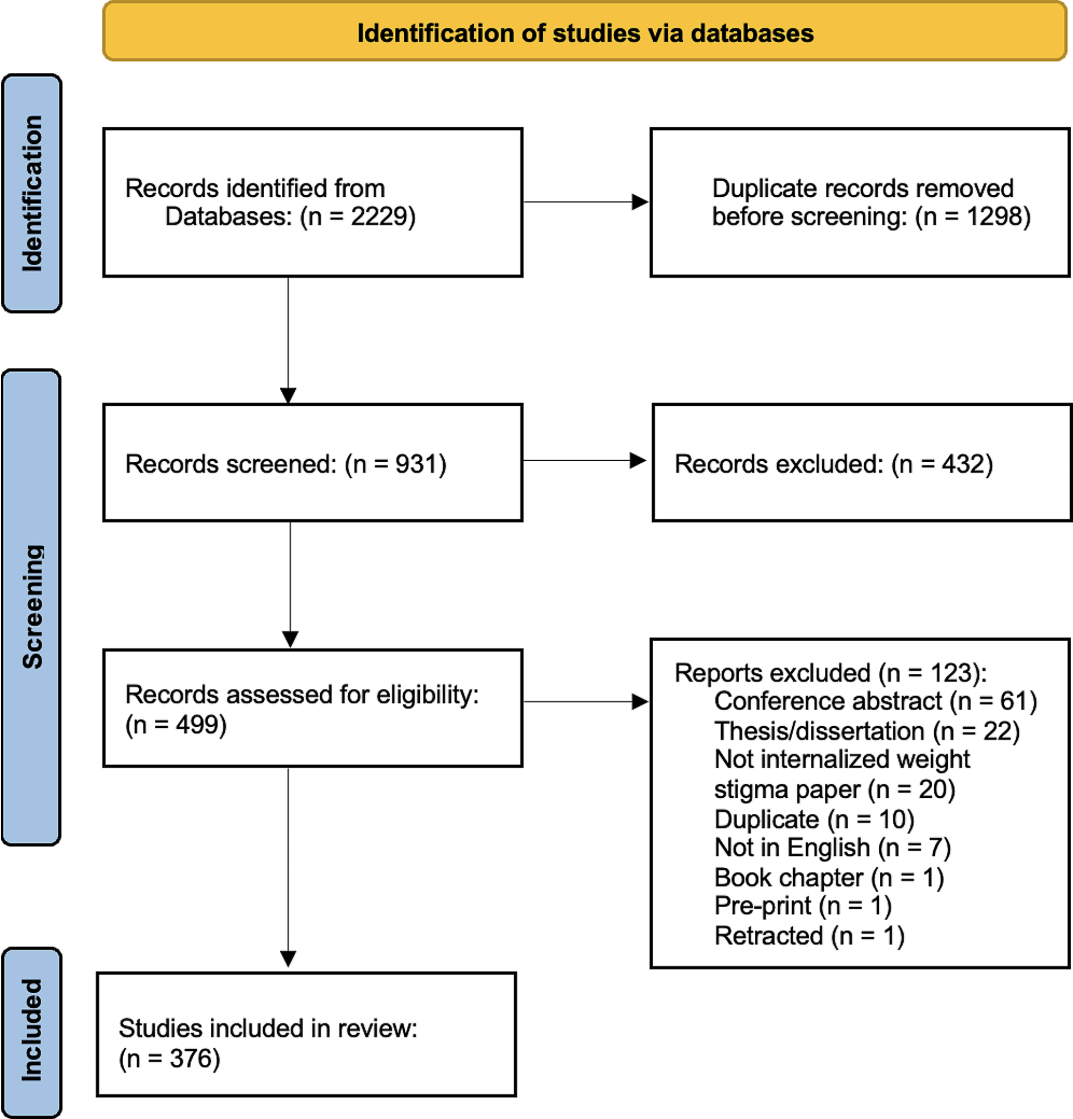
PRISMA Flowchart of Study Selection Process

The search yielded a total of 2,229 records. After the removal of 1,298 duplicates, 931 unique records were screened by the first and second authors (SN, JFS), which resulted in the removal of 432 additional records. The remaining 499 results underwent full-text screening, and an additional 123 records were eliminated. This resulted in 376 studies identified for analysis in the systematic review (see Fig. 1 for the PRISMA flowchart). A reference list of studies included in the review is available in Appendix 1.

### Publication date, publication outlet, Research Location, and Study Methodology

**Fig. 2:**
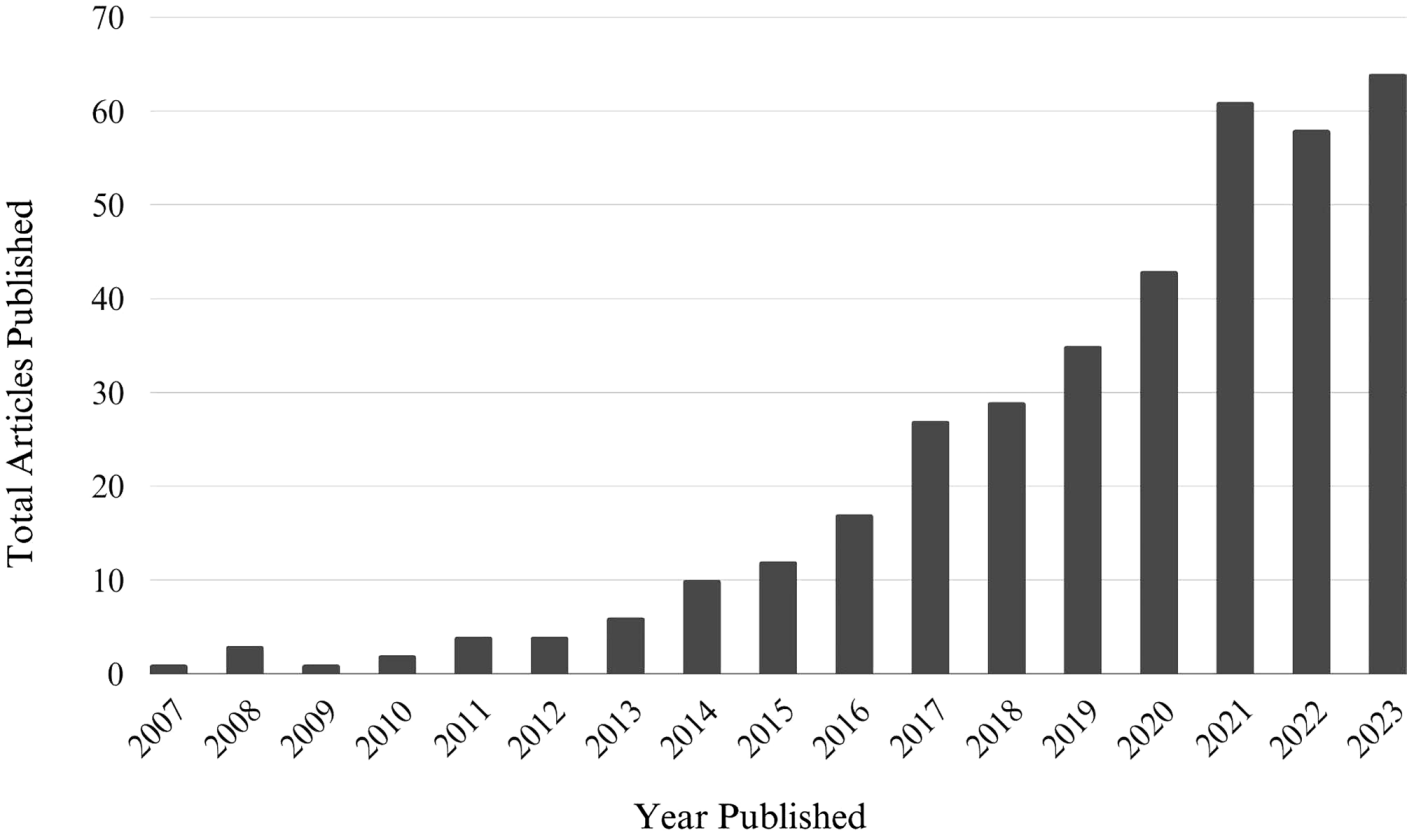
Number of Internalized Weight Stigma Studies Published Per Year

The results of our search indicated that, between 2007 and 2023, 376 articles measuring, reporting, or focusing on internalized weight stigma were published in English in peer-reviewed journals. Figure 2 presents publication by year, highlighting a significant growth in the literature over the last decade. Articles were published in a total of 141 different peer-reviewed journals, summarized in five categories (see Table 1). The body image and eating disorder-specific category included journals that publish exclusively on topics related to body image, eating disorders, and eating behaviours. The psychology-focused category included journals within the broad disciplines of psychology that were not specific to body image or eating disorders. Likewise, the obesity-specific category included journals focused on the topic of obesity, while the healthcare and medical category included journals that were not specific to obesity, but were specific to medical conditions (i.e., diabetes) as well as the provision or evaluation of healthcare. The category of other health-focused included journals that were focused on health but were not specific to obesity, medical conditions, or healthcare. The other category represented journals that did not fall into any of these five categories.

**Table 1:** Summary of Internalized Weight Stigma Publication Outlet

Journal Category	Category Examples	Article Frequency (%)
Body Image and Eating Disorder-Specific	*Eating and Weight Disorders; Eating Disorders; International Journal of Eating Disorders; Journal of Eating Disorders; Body Image, Eating Behaviors, Appetite*	98 (26.06%)
Psychology-Focused	*Frontiers in Psychology; Journal of Health Psychology; Psychology & Health; Psychology of Sexual Orientation and Gender Diversity; Stigma & Health; Journal of Pediatric Psychology; Journal of Contextual Behavioural Science; Mindfulness*	93 (24.73%)
Obesity-Specific	*Clinical Obesity; International Journal of Obesity; Obesity; Obesity Facts; Obesity Research & Clinical Practice; Obesity Reviews; Obesity Science & Practice; Obesity Surgery; Surgery for Obesity and Related Diseases; Pediatric Obesity*	77 (20.48%)
Other Health-Focused	*International Journal of Environmental Research and Public Health; Frontiers in Global Women’s Health; Social Science & Medicine; Nutrients*	58 (15.43%)
Healthcare and Medical	*BMJ Open; Cureus Journal of Medical Science; International Journal of Nursing Practice; Annals of Behavioral Medicine; International Journal of Behavioral Medicine; Journal of Behavioral Medicine*	35 (9.31%)
Other	*PLoS ONE*	15 (3.99%)

*Note* Journals with two or more peer-reviewed studies published on internalized weight stigma are indicated as category examples. Please see Appendix 1 for list of studies included in this review

**Fig. 3:**
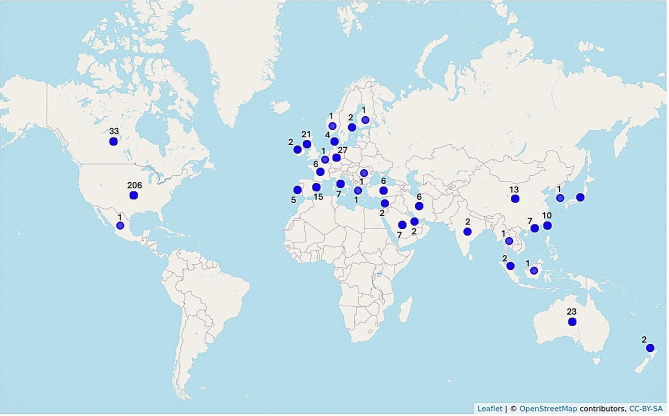
Research Location by Country. *Note.* Fifteen papers in the literature contain multi-national samples, with each country represented in the country total. An additional five papers indicated international samples but did not indicate from which countries data was collected and are, thus, not represented in this figure. For articles that did not report empirical data, the corresponding author’s home country is represented

In examining research location, it is clear that the extant literature has an emerging global evidence base, with 32 countries represented. Figure 3 depicts research representation by country, using the EviAtlas tool for visualizing data [38]. Over half of the literature is represented by researchers and participant samples located in the US (206/376, 54.78%), with the next highest-ranking countries of Canada (33/376, 8.78%), Germany (27/376, 7.18%), Australia (23/376, 6.12%), and United Kingdom (21/376, 5.59%) lagging far behind in geographical representation, as well as little current representation of low- and middle-income countries (10 countries, 33/376 publications, 8.78% of the literature), as per World Bank classifications [39]. The literature is also represented by diverse research methodologies, with a high proportion of cross-sectional studies (240/376, 63.83%) relative to experimental (40/376, 10.64%), qualitative (23/376, 6.12%), and longitudinal research (21/376, 5.58%; see Table 2).

**Table 2:** Methodology of Papers included in review

Methodology	Frequency (%)
Cross-Sectional	240 (63.83%)
Experimental	40 (10.64%)
Qualitative	23 (6.12%)
Longitudinal	21 (5.58%)
Other Review	14 (3.72%)
Systematic Review with or without Meta-Analysis	11 (2.93%)
Commentary or Editorial	8 (2.13%)
Experimental Protocol	6 (1.6%)
Mixed Methods	6 (1.6%)
Ecological Momentary Analysis	4 (1.06%)
Network Analysis	2 (0.53%)
Scoping Review	1 (0.26%)

*Note* The other review category includes all review types that were not indicated as a systematic review (i.e., scoping review, critical review)

### Terms and definitions

**Fig. 4:**
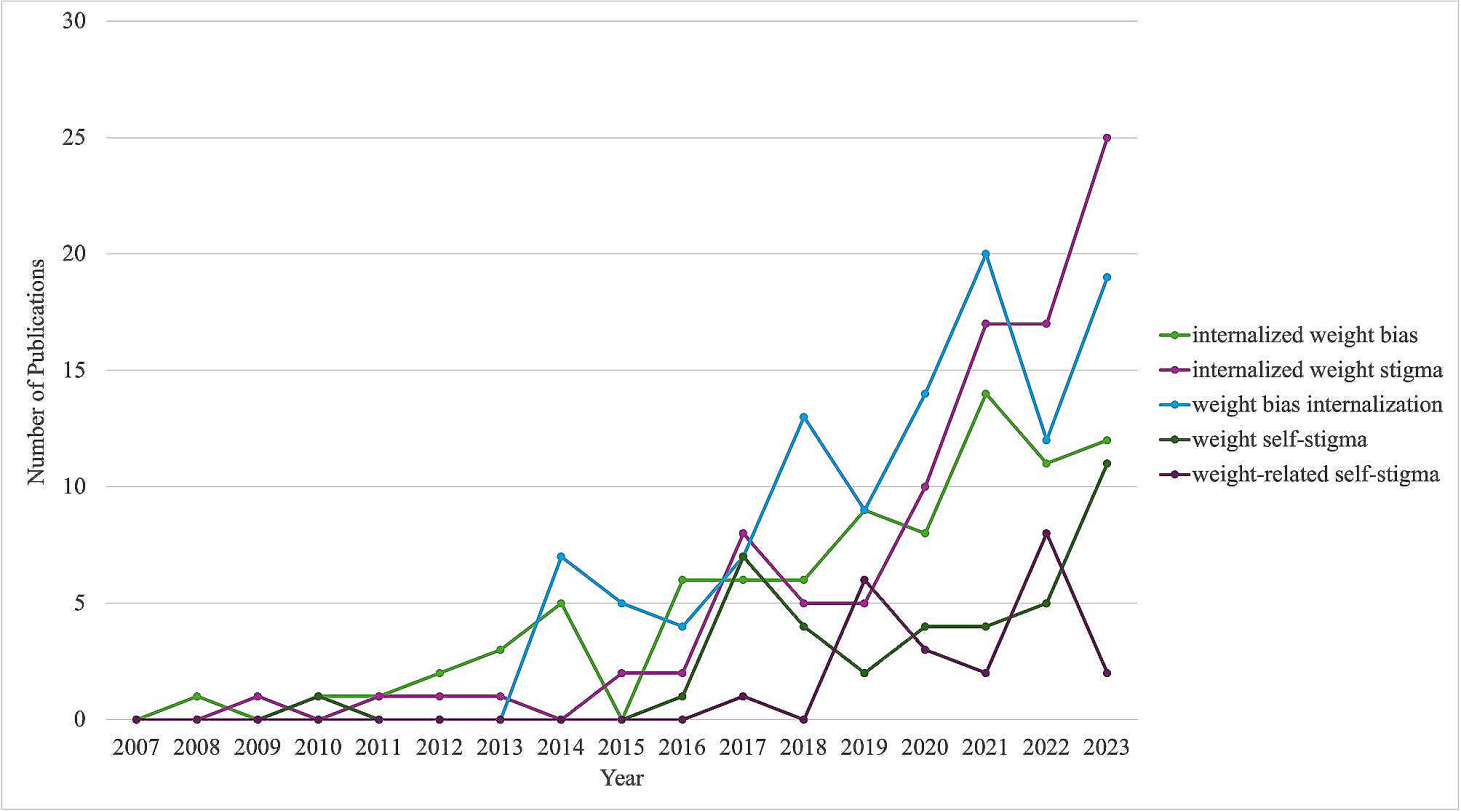
Most Common Terms by Publication Year

Terms used to refer to internalized weight stigma, and their frequencies, are reported in Table 3. As several papers used more than one term to refer to internalized weight stigma, 418 terms were extracted across the 376 papers included in this review. Nearly all papers used a term to refer to internalized weight stigma, with the remaining five papers describing internalized weight stigma without using a specific term to refer to it. Across the literature, 40 unique terms used to refer to internalized weight stigma were identified, with 26 terms used only once. Although many terms used to refer to internalized weight stigma were very similar, with only subtle variations, the exact wording used by researchers was preserved in this analysis, to highlight the variation in terminology across the literature. The three most common terms used in the literature are weight bias internalization (110/418, 26.32%), internalized weight stigma (95/418, 22.73%), and internalized weight bias (85/418, 20.33%). Notably, 26 papers used a non-weight-specific term to refer to internalized weight stigma (i.e., self-stigma, internalized stigma), and seven papers used the terms weight bias, weight stigma, or weight-based stigmatization to refer to internalized weight stigma. The five most common terms used in the literature were plotted across time (Fig. 4), to examine changing trends in terminology use. Specifically, internalized weight stigma appears to have become increasingly popular beginning in 2019, with weight bias internalization and internalized weight bias the second and third most used terms in 2023, respectively.

**Table 3:** Summary of terms used to refer to Internalized Weight Stigma

Term	Frequency (%)
Weight bias internalization	110 (26.32%)
Internalized weight stigma	95 (22.73%)
Internalized weight bias	85 (20.33%)
Weight self-stigma	39 (9.33%)
Weight-related self-stigma	22 (5.26%)
Self-stigma	12 (2.87)
Internalized stigma	5 (1.2%)
Internalization of weight bias	5 (1.2%)
Internalization of weight stigma	3 (0.72%)
Self-directed weight stigma	3 (0.72%)
Internalization of weight-based stereotypes	2 (0.48%)
Internalization of weight stereotypes	2 (0.48%)
Internalized weight-based oppression	2 (0.48%)
Self-stigmatization	2 (0.48%)

*Note* The 14 terms used more than once are indicated in the table. The 26 terms used only once are: body-related self-stigma; indirect individual discrimination; internal weight stigma; internalization of anti-fat attitudes; internalization of bias; internalization of fat hatred; internalization of stereotypes; internalization of stigma; internalized obesity stigma; internalized orthodox obesity messaging; internalized self-stigma; internalized stigma of being fat; internalized stigma related to one’s weight; internalized stigmatization; internalized weight-based oppression; internalized weight-based stereotypes; internalized weight-based stigma; internalized weight-related sigma; internalizing fat stereotypes; internalizing weight bias; self-blame; self-directed stigma; self-directed weight bias; weight stigma internalization; weight-related distress; weight-related self-bias; weight-related self-devaluation

In examining definitions of internalized weight stigma, 252 papers (67.02%) provided a definition. Table 4 summarizes the frequency of the most common components of definitions provided. Similar to our analysis of terms, specificity of language was preserved in this analysis to better understand the subtle variations in definitions of internalized weight stigma. Across the literature, the most common components of definitions of internalized weight stigma were internalized weight-based stereotypes (146/252, 57.93%), internalized negative attitudes or beliefs about weight (104/252, 41.43%), self-devaluation (63/252, 25%), self-blame (17/252, 6.74%), fear of enacted stigma (13/252, 5.16%), negative self-statements, attributions, evaluations, or judgements (12/252, 4.76%), and internalized or perceived weight discrimination (10/252, 3.97%), with many papers defining internalized weight stigma using more than one of these components. When considering the three most common components of definitions, 154 papers (61.1%) defined internalized weight stigma as either internalized stereotypes, internalized negative attitudes or judgement, or self-devaluation; 44 papers (17.46%) included two of these components, and only 13 papers (5.16%) included all three of these components. One paper defined internalized weight stigma as weight stigma (i.e., weight stigma directed towards others).

**Table 4:** Components of definitions of Internalized Weight Stigma

Definition Component	Frequency (%)
Internalized weight-based stereotypes	146 (57.93%)
Internalized negative attitudes or beliefs about weight	104 (41.3%)
Self-devaluation	63 (25%)
Self-blame	17 (6.74%)
Fear of enacted stigma	13 (5.16%)
Negative self-statements, attributions evaluations, or judgements	12 (4.76%)
Internalized or perceived weight discrimination	10 (3.97%)
Poor self-esteem, self-worth, or self-perception	6 (2.38%)
Coping strategy/reaction to weight stigma	4 (1.59%)
Self-shame due to weight	4 (1.59%)
Negative emotions about weight (i.e., guilt)	4 (1.59%)
Belief that one deserves stigma	2 (0.79%)
Self-hate	2 (0.79%)
Acceptance of mistreatment	1 (0.39%)
Belief that weight is due to behaviour	1 (0.39%)
Feeling of low social value	1 (0.39%)
Internalized fear or rejection due to weight stereotypes	1 (0.39%)
Perceived sense of inferiority	1 (0.39%)
Self-alienation due to weight	1 (0.39%)

*Note* Many papers include more than one definition component; Thus, percentages will exceed 100%

## Sample characteristics

Of the 336 articles included in the systematic review that reported original research findings, all but six provided participant demographics (Table 5). Data pertaining to participant age (i.e., adult vs. child/adolescent sample), gender, racial identity, and sexuality was extracted as well as whether or not the sample was exclusively comprised of higher-weight participants, included participants who were actively engaged in weight loss efforts, were involved in pre- or post-operative care for bariatric surgery, or were a sample of participants with a notable clinical characteristic (e.g., pregnancy, eating disorder diagnosis, diabetes diagnosis). Overall, the majority of the literature is represented by samples of non-clinical higher-weight white women.

**Table 5:** Summary of sample characteristics across internalized weight Stigma Research

Sample Characteristic	Frequency (%)
Gender Reported	321/330 (97.27%)
Majority women	228/321 (72.03%)
Included trans/non-binary participants	17/321 (5.3%)
Age Reported	330/330 (100%)
Adult samples	300/330 (90.9%)
Child/adolescent samples	30/330 (9.1%)
Race Reported	205/330 (62.12%)
Majority white participants	141/205 (68.78%)
Sexuality Reported	21/330 (6.36%)
Exclusively higher-weight sample	171/330 (51.82%)
Weight management sample	109/330 (33.03%)
Bariatric surgery sample	25/330 (7.58%)
Other-clinical sample	28/330 (8.48%)

*Note* Of the 376 articles included in the review, 336 reported on empirical research, and 330 reported participant demographics. Samples were indicated as majority women and/or majority white when 60% or more of the sample comprised participants who self-identified as women or white. Relative frequencies based on total reported gender and race reported. All other relative frequencies are indicated based on the total number of articles reporting participant demographics

### Primary Research Topics and General Framing

The diverse research topics represented in the internalized weight stigma literature are summarized in Table 6. Overall, 10 broad categories were identified. A large proportion of the literature is represented by studies examining health-related correlates and outcomes of internalized weight stigma, including psychological health outcomes, physical health outcomes, as well as eating behaviours and eating disorder pathology. The relationship between internalized weight stigma and weight loss or weight control efforts has also received considerable attention in the literature, as has the development, refinement, and translation of measures.

**Table 6:** Summary of primary topics represented in Internalized Weight Stigma Research

Topic Category	Category Examples
Internalized weight stigma	• Correlates and predictors of internalized weight stigma (psychosocial, sociocultural) • Prevalence of internalized weight stigma • Conceptualization of internalized weight stigma
Weight stigma	• Correlate of external weight stigma and related concepts • Internalized weight stigma as an outcome of experienced weight stigma and discrimination
Health outcomes	• Association with physical health outcomes (i.e., cortisol, cardiovascular health, pregnancy and postpartum outcomes, physical activity behaviours and intentions) • Association with mental health outcomes (i.e., psychological distress, suicidality, depression, anxiety, self-esteem) • Correlate of coping responses to experienced weight stigma • Correlate of pre- and post-bariatric surgery outcomes
Body image and eating disorders	• Correlate of eating behaviours, including disordered eating behaviours and eating disorder pathology • Association with symptoms of Binge Eating Disorder • Correlate of body image, body dissatisfaction, and weight perception
Weight loss and management	• Association with total weight loss and maintenance of weight loss in weight loss interventions • Association with weight control behaviours and motivation to engage in weight control behaviours • Correlate of treatment-seeking behaviours and engagement in weight management programs
Healthcare	• Correlate of healthcare avoidance and utilization • Correlate of external weight stigma among healthcare professionals
Interventions for internalized weight stigma	• Outcome of interventions with goal to reduce internalized weight stigma (i.e., acceptance and commitment therapy, cognitive behaviour therapy, mindfulness, self-compassion)
Identity and Intersectionality	• Association with identities, including gender and sexuality • Cross-cultural prevalence and differences in internalized weight stigma
Sociocultural Influences and Impacts	• Association with media use and engagement • Outcome of exposure to advertising and media engagement • Connection to sociocultural discourses about weight and health
Measurement, Psychometrics, and Methods	• Scale development and/or psychometric evaluation • Scale translation

*Note* For each article included in the review 1–2 primary research topics were extracted and summarized into broad topic categories

Finally, each article included in the review was coded for whether or not it was framed around the concept of food addiction, had a focus on weight loss or weight control, had an obesity-focused framing, or had a framing consistent with fat liberation/fat acceptance. Overall, 220/376 papers (58.51%) were identified as having an obesity-focused framing, 113/376 (30.05%) were identified as having a weight control or weight loss focus, and 13 (3.46%) had a food addiction framing. A paper was determined to have an obesity-focused framing when the purpose or significance of the paper was focused on the “obesity epidemic,” the prevalence of obesity, the health consequences of obesity, or a similar obesity-focused framing. Similarly, papers with a purpose or significance related to weight loss or weight control were identified as having a weight loss focus, and papers that measured or notably discussed the topic of food addiction were identified as having a food addiction framing. Numerous papers were rated as having more than one of these framings, with 117 papers (31.12%) rated as having two of the three framings, most often obesity- and weight loss/control-focused, and three papers were rated as having all three framings. Of the remaining papers, 12 (3.19%) had a framing consistent with fat liberation or fat acceptance and 137 (36.44%) were deemed to have a weight-neutral framing. However, it is important to note that, while 137 papers were deemed to have a weight-neutral framing, many of these papers still collected height and weight from participants and reported BMI in their sample demographics, which some may regard as weight-focused.

## Discussion

Our scoping review identified 376 peer-reviewed papers published in English on internalized weight stigma published between 2007 and 2023. We examined and synthesized the literature by publication date, publication outlet, research location, methodology, terminology and definitions used to refer to internalized weight stigma, sample characteristics, primary research topics and general approaches to framing internalized weight stigma research. Our results demonstrate a dramatic growth in research on internalized weight stigma over the last decade, with the majority of the research conducted in the U.S., using cross-sectional methodology, and published in body image/eating disorder journals, followed by psychology-focused and obesity-specific journals. Across the literature, 40 unique terms were used to refer to internalized weight stigma, with weight bias internalization, internalized weight bias, and internalized weight stigma identified as the three most popular terms. Definitions of internalized weight stigma also varied, with 19 unique components identified in the literature. The three most common definition components were internalized weight-based stereotypes, internalized negative attitudes or beliefs about weight, and self-devaluation due to weight. Demographics were reported to varying degrees for most of the original research studies identified in this review, with the majority of samples comprising white, adult, higher-weight women. A wide variety of research topics were identified in the literature, belonging to 10 broad categories, all related to better understanding internalized weight stigma and its associations with weight stigma, health outcomes, body image, eating disorders, and weight loss and management. Finally, the majority of the extant literature is represented by a weight- and obesity-focused framing, including a focus on weight loss, weight control, or food addiction.

Consistent with recent observations, we identified a plethora of terms in the literature to refer to internalized weight stigma, with 26 of 40 terms identified used only once. Austen et al. [14] note that the various terms and definitions used to describe internalized weight stigma are referring to similar, and potentially identical, concepts, but with slight variation. Our results suggest that the majority of unique terms used in the literature are similar, but with slight variations in language, with the majority of researchers using terms that are weight-specific, as opposed to non-specific (i.e., weight self-stigma vs. self-stigma). This variation is similar to the different terms used to refer to weight stigma across the literature (e.g., weight bias, fat phobia, anti-fat stigma, anti-obesity stigma), but with greater variation in research on internalized weight stigma. Similarly, and perhaps more notably, our results confirm the statement by Austen et al. [14], that internalized weight stigma is also defined inconsistently, with 19 unique components identified in definitions across the literature. While we recognize that the definitions used across the literature do generally describe the concept of internalized weight stigma, we argue that the lack of specificity in the language used across definitions and the plethora of variation across the literature incrementally contributes to a lack of conceptual clarity. Without consensus among researchers on a definition of internalized weight stigma, the measurement of internalized weight stigma and the relationship between the components that comprise it will also be inconsistent in the literature, as has been noted in recent work examining the conceptual and measurement overlap between internalized weight stigma and body dissatisfaction [14, 32–35]. This inconsistency will continue to have a ripple effect on the ability of researchers to interpret the meaning and implication of research findings related to internalized weight stigma. Even with a variety in terminology to refer to the concept of internalized weight stigma, a more consistent definition will support enhanced conceptual clarity, measurement, and interpretation of research findings.

In moving towards a consensus on the conceptual definition of internalized weight stigma, it may be important to consider the definitions of other forms of internalized oppression, such as internalized racism. A highly cited definition of internalized racism put forth by Pyke [40] is: “the individual inculcation of the racist stereotypes, values, images, and ideologies perpetuated by the White dominant society about one’s racial group, leading to feelings of self-doubt, disgust, and disrespect for one’s race and/or oneself” [p. 553]. Although helpful, it is important to note that consensus has not been reached on a definition of internalized racism. In describing tensions within internalized racism literature, James [41] noted perceptions among researchers that the concept of internalized racism has been oversimplified as only the internalization of racial stereotypes. In response to perceived oversimplification, Huber et al. [42] defined internalized racism as: “the conscious and unconscious acceptance of a racial hierarchy in which whites are consistently ranked above People of Color…[that] goes beyond the internalization of stereotypes imposed by the white majority…[to] the internalization of the beliefs, values, and worldviews inherent in white supremacy that can potentially result in negative self or racial group perceptions” [p.2]. These two definitions of internalized racism include the three components we identified as most common in the internalized weight stigma literature: internalization of stereotypes, internalization of negative attitudes and beliefs, and self-devaluation. Thus, in moving towards conceptual clarity in the definition of internalized weight stigma, it may be important for researchers to consider a definition that moves beyond the more simplistic internalization of stereotypes, which was the only definition component identified in over 50% of the literature to date. It may also be important for this work to occur alongside a re-examination of instruments used to measure internalized weight stigma, to ensure scale items fully overlap with conceptual definitions.

Our results also highlight an important trend in the topic and framing of research on internalized weight stigma. Together, the health and weight-loss-focused research topics that are common in the literature, as well as the obesity- and weight-focused framing of a substantive portion of the literature, suggest that it may be important to interrogate the extent to which sociocultural discourses on weight and health have influenced the research on internalized weight stigma. Over half of the literature on internalized weight stigma (58.51%) was identified as having a purpose or significance related to obesity or the “obesity epidemic”, and nearly one third of the literature (30.05%) having a purpose or significance related to weight loss or weight control. Further, of the primary research topics identified in the literature, many were weight-focused, such as research examining the health-related correlates of internalized weight stigma (e.g., weight-related quality of life, lifestyle behaviours and intentions), research on weight loss and weight control (e.g., internalized weight stigma as a barrier to engagement in weight loss programs or as associated with lower total weight loss), and research on disordered eating and eating disorders (e.g., greater focus on binge eating and binge eating disorder relative to restrictive eating behaviours).

The extent to which the literature on internalized weight stigma is framed alongside obesity, weight loss, and weight control is concerning, as it highlights a weight-centric approach to research that may unintentionally reinforce sociocultural discourses about weight and health, also known as diet culture. Diet culture is defined as encompassing: (1) food and eating myths that moralize food choices; (2) myths that weight is a reliable proxy for health; (3) a moral hierarchy of bodies in society that idealize thinness and demonize fatness; and (4) connections to other forms of systemic oppression, including racism, patriarchy, and capitalism [43]. This definition of diet culture is consistent with what Rodgers [44] described as “healthy weight discourse”. Unique from, but related to, sociocultural discourses that emphasize thinness, healthy weight discourse emphasizes that lower body weights are the healthiest, that weight is easily controllable, and that weight is a moral responsibility.^1^ Although research on internalized weight stigma may not directly endorse such discourses, the weight- and obesity-focused framings in the literature may do so unintentionally. For example, the examination of internalized weight stigma as a barrier to weight loss or engagement in weight loss efforts may unintentionally reinforce assumptions that weight is a proxy for health or that weight is an individual responsibility, as well as morality discourses associated with food choices and body size. Further, a focus on binge eating behaviours and binge eating disorder, at the expense of other forms of disordered eating may unintentionally reinforce stereotypes about the eating behaviours of higher-weight people (i.e., gluttonous, overeating) as well as stereotypes about what different eating disorders ‘look like’ (i.e., higher-weight people as having Binge Eating Disorder and lower-weight people having Anorexia Nervosa or Bulimia Nervosa). The consequences of stereotypes about the presentation of eating disorders on healthcare for higher-weight people with restrictive eating disorders are beginning to receive attention in the literature [45, 46]. We believe that the significant effort of researchers to document the health consequences of internalized weight stigma is a strength of the literature. However, when these health consequences are directly connected to obesity or weight-loss framings (e.g., weight-related quality of life, lifestyle behaviours, internalized weight stigma as a barrier to weight loss, binge eating behaviour), it becomes difficult to parse out the health consequences associated with weight stigma from potentially stigmatizing sociocultural discourses about weight and health.

In describing this potential for unintentional harm, we do not intend to advocate for the cessation of research related to the impact of internalized weight stigma on physical and psychological health. We also do not intend to declare that all internalized weight stigma research that references obesity is explicitly stigmatizing or blames higher-weight people for their experiences. Rather, we aim to identify the potential unintended consequences of a weight-centric approach to research on internalized weight stigma that frames internalized weight stigma around the ‘problem of obesity’, weight, and weight loss. Instead, we call for a weight-neutral approach, such as introducing internalized weight stigma through the concept of weight stigma or in such a way that de-centers a focus on weight or negative health outcomes associated with higher body weights. In describing the consequences of a weight-centric approach to healthcare, Mauldin et al. [47] proposed a series of recommendations for engaging in weight-inclusive practice. These recommendations included: (1) not pathologizing individuals based on weight or BMI; (2) reducing a focus on weight in practice; (3) asking about, and optimizing, healthy behaviours in all individuals in a shame-free context; and (4) recognizing weight stigma as well as challenging personal assumptions about weight. In research, reducing a focus on weight may mean that researchers identify alternative outcome measures that are more accurate health indicators. The recommendations by Mauldin at al. [47] for a weight-inclusive approach to healthcare are consistent with recent recommendations for changing obesity narratives in global research and healthcare practices to reduce weight stigma [48]. These recommendations included important considerations for language use, including eliminating the use of alarmist or catastrophizing language (i.e., “obesity epidemic”, “war on obesity”) and distinguishing between body size and obesity (i.e., not pathologizing individuals solely based on BMI) [48].

Finally, our scoping review provided an opportunity to investigate what populations are over- and under-represented in the literature. Although nearly all empirical studies reported demographics, the gender and age of samples were the only identities consistently reported in the literature (97.4% and 100%, respectively). Race was reported in only 64% of papers, sexuality in 5.2%, and transgender or nonbinary gender identities in 2.3% of papers. Although racial, sexuality, and gender-based differences may not be a primary focus in the analyses of research, reporting these identities consistently is important to transparently report who is represented in the findings of a study as well as for identifying priorities for increasing the representation of diverse research samples in the literature. Our results suggest that research on internalized weight stigma lacks diversity, as most of the research is characterized by samples of adults, women, white participants, and higher-weight participants. Further, together with the data on research location, the literature is over-represented by samples of people from Western, Educated, Industrialized, Rich and Democratic (WEIRD) societies [49]. These findings are consistent with what Landor et al. [50] describe as a manifestation of white supremacy in body image and eating disorders research, whereby the experiences of middle- and upper-class white women were historically prioritized alongside assumptions that body image disturbance did not occur among people of colour, with cascading consequences for current body image research. Increasing the diversity of participant samples and the global representation (i.e., race/ethnicity, gender spectrum, sexuality, body size, socioeconomic status, (dis)ability, geographical location) in future research will support a more accurate understanding of internalized weight stigma within and across populations. Such efforts in future research will ensure a more complete understanding of the experience and impact of internalized weight stigma, including the impact of internalized weight stigma on marginalized individuals, ensuring research findings are reflective of every body [50].

### Strengths, limitations, and future research directions

Several strengths and limitations of the current research are important to note. First, a strength of this paper is its comprehensiveness, as this is the first review of the entire literature on internalized weight stigma. Given the nature of this review, our findings may support ongoing discussions surrounding conceptual and measurement clarity, as well as provide directions for future work in this area. Second, our methods included a comprehensive review of several databases, to conduct as complete a review as possible. However, this work is not without its limitations. Our review is inherently incomplete due to the exclusion of publications published in languages other than English. Further, it was beyond the scope of the paper to utilize more in-depth methodology for extracting and analyzing data, especially data pertaining to definitions of internalized weight stigma, research topic and journal type. Rather, our synthesis was based on simple thematic/content categorization to summarize definition components, primary research topics, and journals. To further the scholarly dialogue on conceptual clarity, a more meaningful thematic analysis of definitions may be useful. Our synthesis of primary research topics was further limited by the extraction of only 1–2 primary topics per paper. Thus, this synthesis did not allow for a detailed and nuanced description of all major concepts examined in the literature and serves as only a cursory description.

Despite these limitations, there are several key takeaways from this research that can support future research on internalized weight stigma. These research directions include:

1. Engaging in continued efforts to enhance the conceptual clarity of internalized weight stigma. These efforts may involve a Delphi study, as previously suggested [14] to develop consensus on preferred terminology and definitions for internalized weight stigma. Researchers may wish to consider the inclusion of people with lived experience of internalized weight stigma as experts to be consulted in a Delphi study.
2. Considering ways to engage in weight-neutral research on internalized weight stigma by reducing or eliminating obesity and weight-loss research framings. This may involve considering research outcomes other than weight loss and BMI in study methodology and communicating about research through a framing other than obesity. When research is focused on health conditions and outcomes associated with higher-weights, it is important to consider how to engage in this research in ways that do not unintentionally reinforce weight-based stereotypes and diet culture myths. This may include reducing the emphasis on internalized weight stigma as a barrier to weight loss. For research that investigates internalized weight stigma in the context of disordered eating behaviours and eating disorders, researchers are encouraged to also include measures of restrictive eating and purging behaviours, to ensure a more complete screening of eating disorder symptoms is conducted.
3. Examining internalized weight stigma in under-represented areas of the literature. While research examining the health, eating, and body image correlates of internalized weight stigma has grown, there are fewer studies examining internalized weight stigma in the context of: (a) healthcare utilization; (b) experienced weight stigma in healthcare; (c) the connection of internalized weight stigma to broader sociocultural discourses related to food, weight, and appearance; (d) the influence of media, particularly social media, on internalized weight stigma; (e) as well as the developmental trajectory of internalized weight stigma across the lifespan. We recognize that these are only a few of the many potential future research directions.
4. Diversify research methodologies. Given that the majority of the research is represented by cross-sectional research, an increase in other methodologies may support an enhanced understanding of internalized weight stigma. Researchers are encouraged to consider longitudinal, experimental, and qualitative designs, among other methodological approaches.
5. Contribute to an increasingly diverse and global literature on internalized weight stigma. Researchers are encouraged to provide more detailed demographic details of research participants, and to seek out participants that are currently underrepresented in the literature. Researchers are also encouraged to increase the evidence base among child and adolescent samples as well as among participants that are not from WEIRD societies, including research conducted in low- and middle-income countries.

## Conclusion

Our scoping review of the internalized weight stigma literature suggests this area of research is quickly growing and that researchers are investigating internalized weight stigma alongside a wide variety of topics and concepts. Overall, this literature has significantly contributed to our understanding of the impact of systemic weight stigma in society on physical and psychological health outcomes. Our synthesis of the literature confirms a lack of concept clarity, based on the lack of consistency in terminology and definitions of internalized weight stigma and provides considerations for moving forward. It also highlights the potential unintended consequences of research conducted from an obesity-focused or weight loss-focused framing. We encourage researchers to consider a weight-neutral framing moving forward, to decrease the risk of unintentional perpetuation of weight stigma in research. Overall, furthering conceptual clarity and measurement as well as continuing to fill the gaps in the internalized weight stigma literature from a weight-neutral perspective is critically important to better understanding the impact of internalized weight stigma on the lives of individuals across the weight spectrum globally.

### Electronic supplementary material

Below is the link to the electronic supplementary material.

**Supplementary Material 1**. Appendix 1

## Data Availability

The extracted data and analysis file for the current study are available from the corresponding author upon reasonable request.

## Footnotes

1 A review of research that supports the components of diet culture and healthy weight discourse as myths is beyond the scope of this paper. The authors recommend that interested readers begin by reading Flegal’s (2021) chapter in the *Routledge Handbook of Critical Obesity Studies* and Li et al., (2021) *PLoS ONE*, in addition to those articles cited in this section of the paper.
