# Supplementary material for: Current trends and future directions in internalized weight stigma research: a scoping review and synthesis of the literature
Source: J Eat Disord. 2024 Jul 15;12:98. doi: 10.1186/s40337-024-01058-0 (PMC11247756; doi:10.1186/s40337-024-01058-0)
Supplement: Supplementary file 1 — Supplementary Material 1. Appendix 1 [file 40337_2024_1058_MOESM1_ESM.docx]

**Appendix 1**

**APA7 Reference List of Articles Included in the Systematic Review**

Alswat, K. A., Waslallah Alsuwat, R., Metaeb Aljaed, K., & Almalki, D. A. M. (2020). Prevalence and impact of weight self stigmatization on type II diabetes glycemic and cardiovascular markers control. *Pakistan Journal of Biological Sciences*, *23*(10), 1285–1290. <https://doi.org/10.3923/pjbs.2020.1285.1290>

Ahorsu, D. K., Lin, C. Y., Imani, V., Griffiths, M. D., Su, J. A., Latner, J. D., Marshall, R. D., & Pakpour, A. H. (2020). A prospective study on the link between weight-related self-stigma and binge eating: Role of food addiction and psychological distress. *International Journal of Eating Disorders*, *53*(3), 442–450. <https://doi.org/10.1002/eat.23219>

Aimé, A., Fuller-Tyszkiewicz, M., Dion, J., Markey, C. H., Strodl, E., McCabe, M., Mellor, D., Granero Gallegos, A., Pietrabissa, G., Alcaraz-Ibánez, M., Bégin, C., Blackburn, M. È., Caltabiano, M., Castelnuovo, G., Gullo, S., Hayami-Chisuwa, N., He, Q., Lo Coco, G., Manzonie, G. M., … Maïano, C. (2020). Assessing positive body image, body satisfaction, weight bias, and appearance comparison in emerging adults: A cross-validation study across eight countries. *Body Image*, *35*, 320–332. <https://doi.org/10.1016/j.bodyim.2020.09.014>

Aladel, A., Dakhakhni, B., Almuhtadi, Y., Alsheweir, A., & Aljammaz, S. (2023). Effect of weight self-stigma on quality of life and dietary habits among adult students in Riyadh, Saudi Arabia. *Healthcare*, *11*(12), 1754. <https://doi.org/10.3390/healthcare11121754>

Alberga, A. S., Russell-Mayhew, S., von Ranson, K. M., & McLaren, L. (2016). Weight bias: A call to action. *Journal of Eating Disorders*, *4*, 34. <https://doi.org/10.1186/s40337-016-0112-4>

Aldhahi, M. I., Al Khalil, W. K., Almutiri, R. B., Alyousefi, M. M., Alharkan, B. S., & AnNasban, H. (2022). Effect of weight self-stigma and self-esteem on aerobic exercise capacity in adult women with different body compositions. *International Journal of Environmental Research and Public Health*, *19*(2). <https://doi.org/10.3390/ijerph19020873>

Alimoradi, Z., Golboni, F., Griffiths, M. D., Broström, A., Lin, C.-Y., & Pakpour, A. H. (2020). Weight-related stigma and psychological distress: A systematic review and meta-analysis. *Clinical Nutrition*, *39*(7), 2001–2013. <https://doi.org/10.1016/j.clnu.2019.10.016>

Almenara, C., Aime, A., Maiano, C., Ejova, A., Guevremont, G., Bournival, C., & Ricard, M. (2017). Weight stigmatization and disordered eating in obese women: The mediating effects of self-esteem and fear of negative appearance evaluation. *European Review of Applied Psychology*, *67*(3), 155–162. <https://doi.org/10.1016/j.erap.2017.02.004>

Almutairi, B. F., Alsaygh, K. W., Altamimi, M. M., Alshammari, A. S., Alsomali, A. M., Alanazi, S. M., Alzahrani, S. M., Alsaad, A. S., & Zacharakis, G. (2021). Internalized weight stigma: Prevalence and association with psychiatric disorder among overweight and obese individuals. *Cureus*, *13*(10), e18577. <https://doi.org/10.7759/cureus.18577>

Althumiri, N. A., Basyouni, M. H., AlMousa, N., AlJuwaysim, M. F., Alhamdan, A. A., Al-Qahtani, F. S., BinDhim, N. F., & Alqahtani, S. A. (2021). Exploring weight stigma in Saudi Arabia: A nationwide cross-sectional study. *International Journal of Environmental Research and Public Health*, *18*(17). <https://doi.org/10.3390/ijerph18179141>

Ananthakumar, T., Jones, N. R., Hinton, L., & Aveyard, P. (2020). Clinical encounters about obesity: Systematic review of patients’ perspectives. *Clinical Obesity*, *10*(1), e12347. <https://doi.org/10.1111/cob.12347>

Anastasiadou, D., Slater, M., Spanlang, B., Cano Porras, D., Comas, M., Ciudin, A., Puig, G. P., Vazquez-De Sebastian, J., Ramos-Quiroga, J. A., & Lusilla-Palacios, P. (2022). Clinical efficacy of a virtual reality tool for the treatment of obesity: Study protocol of a randomised controlled trial. *BMJ Open*, *12*(6), e060822. <https://doi.org/10.1136/bmjopen-2022-060822>

Andres, A., Fornieles-Deu, A., Sepulveda, A. R., Beltran-Garrayo, L., Montcada-Ribera, A., Bach-Faig, A., & Sanchez-Carracedo, D. (2022). Spanish validation of the Modified Weight Bias Internalization Scale (WBIS-M) for adolescents. *Eating and Weight Disorders*, *27*(8), 3245–3256. <https://doi.org/10.1007/s40519-022-01453-z>

Arguelles, D., Perez-Samaniego, V., & Lopez-Canada, E. (2021). “Do you find it normal to be so fat?” Weight stigma in obese gym users. *International Review for the Sociology of Sport*, *57*(7)*.* <https://doi.org/10.1177/10126902211056867>

Argyrides, M., Charalambous, Z., Anastasiades, E., & Michael, K. (2022). Translation and validation of the Greek version of the Modified Weight Bias Internalization Scale in an adult population. *Clinical Obesity*, *12*(2), e12503. <https://doi.org/10.1111/cob.12503>

Austen, E., Greenaway, K. H., & Griffiths, S. (2020). Differences in weight stigma between gay, bisexual, and heterosexual men. *Body Image*, *35*, 30–40. <https://doi.org/10.1016/j.bodyim.2020.08.002>

Austen, E. & Griffiths, S. (2022). Weight stigma predicts reduced psychological wellbeing and weight gain among sexual minority men: A 12-month longitudinal cohort study using random intercept cross-lagged panel models. *Body Image*, *40*, 19–29. <https://doi.org/10.1016/j.bodyim.2021.10.006>

Austen, E., Pearl, R. L., & Griffiths, S. (2021). Inconsistencies in the conceptualisation and operationalisation of internalized weight stigma: A potential way forward. *Body Image*, *36*, iii–v. <https://doi.org/10.1016/j.bodyim.2020.12.002>

Baldofski, S., Rudolph, A., Tigges, W., Herbig, B., Jurowich, C., Kaiser, S., Dietrich, A., & Hilbert, A. (2016). Weight bias internalization, emotion dysregulation, and non-normative eating behaviors in prebariatric patients. *International Journal of Eating Disorders*, *49*(2), 180–185. <https://doi.org/10.1002/eat.22484>

Barber, J. A., Palmese, L., Reutenauer, E. L., Grilo, C. M., & Tek, C. (2011). Implications of weight-based stigma and self-bias on quality of life among individuals with schizophrenia. *The Journal of Nervous Mental Disease*, *199*(7), 431–435. <https://doi.org/10.1097/NMD.0b013e318221403d>

Barlosius, E., & Philipps, A. (2015). Felt stigma and obesity: Introducing the generalized other. *Social Science & Medicine*, *130*, 9–15. <https://doi.org/10.1016/j.socscimed.2015.01.048>

Barnhart W.R., Cui S., Cui T., & He J. (2023). Relationships between weight bias internalization and biopsychosocial health outcomes: A prospective study in Chinese adolescents. *International Journal of Eating Disorders*, *56*(5), 1021–1033. <https://doi.org/10.1002/eat.23904>

Barnhart, W. R., Cui, T., Cui, S., Ren, Y., Ji, F., & He, J. (2023). Exploring the prospective relationships between food addiction symptoms, weight bias internalization, and psychological distress in Chinese adolescents. *International Journal of Eating Disorders*, *56*(12), 2304–2314. <https://doi.org/10.1002/eat.24066>

Baur, J., Schmitz, F., Naumann, E., & Svaldi, J. (2022). Implicit attitudes towards weight, one’s own body and its relation to food in women with overweight and obesity. *Cognitive Therapy and Research*, *46*(2), 436–447. <https://doi.org/10.1007/s10608-021-10271-z>

Becker, C. B., Middlemas, K., Gomez, F., & Kilpela, L. S. (2021). An exploratory examination of internalized weight stigma in a sample living with food insecurity. *Body Image*, *37*, 238–245. <https://doi.org/10.1016/j.bodyim.2021.03.006>

Becker, C. B., Middlemass, K., Taylor, B., Johnson, C., & Gomez, F. (2017). Food insecurity and eating disorder pathology. *International Journal of Eating Disorders*, *50*(9), 1031–1040. <https://doi.org/10.1002/eat.22735>

Beltrán-Garrayo, L., Rojo, M., Rodríguez-Mondragón, L., & Sepúlveda García, A. R. (2023). Weight bias internalization among adolescents in Spain: Psychological correlates across gender diversity and weight status. *Revista de Psicología Clínica Con Niños y Adolescentes*, *10*(2), 1–8. <https://doi.org/10.21134/rpcna.2023.10.1.2>

Bennell K. L., Jones S. E., Hinman R. S., McManus F., Lamb K. E., Quicke J. G., Sumithran P., Prendergast J., George E. S., Holden M. A., Foster N. E., & Allison K. (2022). Effectiveness of a telehealth physiotherapist-delivered intensive dietary weight loss program combined with exercise in people with knee osteoarthritis and overweight or obesity: Study protocol for the POWER randomized controlled trial. *BMC Musculoskeletal Disorders*, *23*(1), 733. <https://doi.org/10.1186/s12891-022-05685-z>

Bennett, B. L., Lawson, J. L., Funaro, M. C., & Ivezaj, V. (2022). Examining weight bias before and/or after bariatric surgery: A systematic review. *Obesity Reviews*, *23*(11), e13500. <https://doi.org/10.1111/obr.13500>

Bennett, B. L., Wagner, A. F., & Latner, J. D. (2022). Body checking and body image avoidance as partial mediators of the relationship between internalized weight bias and body dissatisfaction. *International Journal of Environmental Research and Public Health*, *19*(16), 9785. <https://doi.org/10.3390/ijerph19169785>

Bernard, M., Lobner, M., Lordick, F., Mehnert-Theuerkauf, A., Riedel-Heller, S. G., & Luck-Sikorski, C. (2022). Cancer prevention in females with and without obesity: Does perceived and internalised weight bias determine cancer prevention behaviour? *BMC Women’s Health*, *22*(1), 511. <https://doi.org/10.1186/s12905-022-02085-2>

Bessey, M., Brady, J., Lordly, D., & Leighteizer, V. (2021). “This is what you’re supposed to do”: Weight stigma in dietetics education. *Fat Studies*, 10(2), 184–196. <https://doi.org/10.1080/21604851.2020.1859078>

Bevan, N., O’Brien, C. K. S., Latner, J. D., Vandenberg, B., Jeanes, R., & Lin, C.-Y. (2023). The relationship between weight stigmatization, avoidance, enjoyment and participation in physical activity and sport, and psychological distress. *American Journal of Health Behavior*, *47*(2), 360–368. <https://doi.org/10.5993/AJHB.47.2.15>

Bevan, N., O’Brien, K. S., Lin, C. Y., Latner, J. D., Vandenberg, B., Jeanes, R., Puhl, R. M., Chen, I. H., Moss, S., & Rush, G. (2021). The relationship between weight stigma, physical appearance concerns, and enjoyment and tendency to avoid physical activity and sport. *International Journal of Environmental Research and Public Health*, *18*(19). <https://doi.org/10.3390/ijerph18199957>

Bidstrup, H., Brennan, L., Hindle, A., Kaufmann, L., & De La Piedad Garcia, X. (2022). Internalised weight stigma mediates relationships between perceived weight stigma and psychosocial correlates in individuals seeking bariatric surgery: A cross-sectional study. *Obesity Surgery,* *32*(11), 3675–3686. <https://doi.org/10.1007/s11695-022-06245-z>

Bidstrup, H., Brennan, L., Kaufmann, L., & de la Piedad Garcia, X. (2022). Internalised weight stigma as a mediator of the relationship between experienced/perceived weight stigma and biopsychosocial outcomes: A systematic review. *International Journal of Obesity*, *46*(1), 1–9. <https://doi.org/10.1038/s41366-021-00982-4>

BinDhim, N. F., Althumiri, N. A., Basyouni, M. H., Sims, O. T., Alhusseini, N., & Alqahtani, S. A. (2020). Arabic Translation of the Weight Self-Stigma Questionnaire: Instrument validation study of factor structure and reliability. *JMIR Formative Research*, *4*(11), e24169. <https://doi.org/10.2196/24169>

Bombak, A. (2015). “Everybody watches and everybody comments” Health-at-Every-Size and dieting in a fat-phobic world. *Food Culture & Society*, *18*(4), 681–700. <https://doi.org/10.1080/15528014.2015.1088196>

Boswell, R. G. & White, M. A. (2015). Gender differences in weight bias internalisation and eating pathology in overweight individuals. *Advances in Eating Disorders*, *3*(3), 259–268. <https://doi.org/10.1080/21662630.2015.1047881>

Braun, T. D., Gorin, A. A., Puhl, R. M., Stone, A., Quinn, D. M., Ferrand, J., Abrantes, A. M., Unick, J., Tishler, D., & Papasavas, P. (2021). Shame and self-compassion as risk and protective mechanisms of the internalized weight bias and emotional eating link in individuals seeking bariatric surgery. *Obesity Surgery*, *31*(7), 3177–3187. <https://doi.org/10.1007/s11695-021-05392-z>

Braun, T. D., Olson, K., Panza, E., Lillis, J., Schumacher, L., Abrantes, A. M., Kunicki, Z., & Unick, J. L. (2022). Internalized weight stigma in women with class III obesity: A randomized controlled trial of a virtual lifestyle modification intervention followed by a mindful self-compassion intervention. *Obesity Science and Practice*, *8*(6), 816–827. <https://doi.org/10.1002/osp4.616>

Braun, T. D., Puhl, R. M., Quinn, D. M., Gorin, A., Tishler, D., & Papasavas, P. (2022). Weight stigma and posttraumatic stress disorder symptoms in individuals seeking bariatric surgery. *Surgery for Obesity and Related Diseases*, *18*(8), 1066–1073. <https://doi.org/10.1016/j.soard.2022.05.011>

Braun, T. D., Riley, K. E., Kunicki, Z. J., Finkelstein-Fox, L., Conboy, L. A., Park, C. L., Schifano, E., Abrantes, A. M., & Lazar, S. W. (2021). Internalized weight stigma and intuitive eating among stressed adults during a mindful yoga intervention: Associations with changes in mindfulness and self-compassion. *Health Psychology and Behavioral Medicine*, *9*(1), 933–950. <https://doi.org/10.1080/21642850.2021.1992282>

Braun, T. D., Unick, J. L., Abrantes, A. M., Dalrymple, K., Conboy, L. A., Schifano, E., Park, C. L., & Lazar, S. W. (2022). Intuitive eating buffers the link between internalized weight stigma and body mass index in stressed adults. *Appetite*, *169*, 105810. <https://doi.org/10.1016/j.appet.2021.105810>

Brenton-Peters, J. M., Consedine, N. S., Cavadino, A., Roy, R., & Serlachius, A. S. (2022). Investigating the effect of an online self-compassion for weight management (SC4WM) intervention on self-compassion, eating behaviour, physical activity and body weight in adults seeking to manage weight: Protocol for a randomised controlled trial. *BMJ Open*, *12*(2), e056174. <https://doi.org/10.1136/bmjopen-2021-056174>

Brochu, P., Veillette, L., Serrano, J., & Seidl, M. (2021). It’s interpersonal: Internalized weight bias and suicidality are associated indirectly via perceived burdensomeness and thwarted belongingness. *Stigma and Health*, *6*(3), 287–295. <https://doi.org/10.1037/sah0000264>

Brown, A., & Flint S. W. (2021). Preferences and emotional response to weight-related terminology used by healthcare professionals to describe body weight in people living with overweight and obesity. *Clinical Obesity*, *11*(5), e12470. <https://doi.org/10.1111/cob.12470>

Brownstone, L. M., Kelly, D. A., Ko, S.-J., Jasper, M. L., Sumlin, L. J., Hall, J., Tiede, E., Dinneen, J., Anderson, E., & Goffredi, A. R. (2021). Dismantling weight stigma: A group intervention in a partial hospitalization and intensive outpatient eating disorder treatment program. *Psychotherapy*, *58*(2), 282–287. <https://doi.org/10.1037/pst0000358>

Brun, I., Russell-Mayhew, S., Klingle, K., & Nutter, S. (2021). Overt restrictive feeding for weight management: A preliminary retrospective examination of childhood experiences. *Eating and Weight Disorders*, *26*(7), 2407–2411. <https://doi.org/10.1007/s40519-020-01036-w>

Burmeister, J. M., & Carels, R. A. (2014). Television use and binge eating in adults seeking weight loss treatment. *Eating Behaviors*, *15*(1), 83–86. <https://doi.org/10.1016/j.eatbeh.2013.10.001>

Burmeister, J. M., Hinman, N., Koball, A., Hoffmann, D. A., & Carels, R. A. (2013). Food addiction in adults seeking weight loss treatment. Implications for psychosocial health and weight loss. *Appetite*, *60*(1), 103–110. <https://doi.org/10.1016/j.appet.2012.09.013>

Burnette, C. B., & Mazzeo, S. E. (2020). An uncontrolled pilot feasibility trial of an intuitive eating intervention for college women with disordered eating delivered through group and guided self-help modalities. *International Journal of Eating Disorders*, *53*(9), 1405–1417. <https://doi.org/10.1002/eat.23319>

Burnette, C. B., & Mazzeo, S. E. (2020). Examining the contribution of weight-bias internalization to the associations between weight suppression and disordered eating in undergraduates. *Eating Behaviors*, *37*, 101392. <https://doi.org/10.1016/j.eatbeh.2020.101392>

Butt, M., Harvey, A., Khesroh, E., Rigby, A., & Paul, I. M. (2023). Assessment and impact of paediatric internalized weight bias: A systematic review. *Pediatric Obesity*, *18*(7), e13040. <https://doi.org/10.1111/ijpo.13040>

Butt, M., Su, L., & Rigby, A. (2022). Associations of use of social media and psychopathology and body image in pre- and post-surgical bariatric samples: A cross-sectional analysis. *Obesity Surgery*, *32*(9), 3047–3055. <https://doi.org/10.1007/s11695-022-06206-6>

Calugi, S., & Dalle Grave, R. (2020). Psychological features in obesity: A network analysis. *International Journal of* *Eating Disorders*, *53*(2), 248–255. <https://doi.org/10.1002/eat.23190>

Calugi, S., Segattini, B., Cattaneo, G., Chimini, M., Dalle Grave, A., Dametti, L., Molgora, M., & Dalle Grave, R. (2023). Weight bias internalization and eating disorder psychopathology in treatment-seeking patients with obesity. *Nutrients*, *15*(13), 2932. <https://doi.org/10.3390/nu15132932>

Carels, R., Hlavka, R., Selensky, J., Solar, C., Rossi, J., Miller, J., & Ellis, J. (2020). The Associations between wives’ internalized weight bias and other weight-related concerns, perceived husbands’ weight-related comments, perceived mate value, and psychological and relationship outcomes. *Stigma and Health*, *5*(3), 258–268. <https://doi.org/10.1037/sah0000192>

Carels, R., Miller, J., Selensky, J., Hlavka, R., Solar, C., Rossi, J., & Ellis, J. (2019). Using an acceptance-based behavioral approach as a supplement to obesity treatment: A stepped-care approach. *Journal of Contextual Behavioral Science*, *12*, 98–105. <https://doi.org/10.1016/j.jcbs.2019.03.002>

Carels, R. A., Burmeister, J., Oehlhof, M. W., Hinman, N., LeRoy, M., Bannon, E., Koball, A., & Ashrafloun, L. (2013). Internalized weight bias: Ratings of the self, normal weight, and obese individuals and psychological maladjustment. *Journal of Behavioral Medicine*, *36*(1), 86–94. <https://doi.org/10.1007/s10865-012-9402-8>

Carels, R. A., Burmeister, J. M., Koball, A. M., Oehlhof, M. W., Hinman, N., LeRoy, M., Bannon, E., Ashrafioun, L., Storfer-Isser, A., Darby, L. A., & Gumble, A. (2014). A randomized trial comparing two approaches to weight loss: Differences in weight loss maintenance. *Journal of Health Psychology*, *19*(2), 296–311. <https://doi.org/10.1177/1359105312470156>

Carels, R. A., Hinman, N., Koball, A., Oehlhof, M. W., Gumble, A., & Young, K. M. (2011). The self-protective nature of implicit identity and its relationship to weight bias and short-term weight loss. *Obesity Facts*, *4*(4), 278–283. <https://doi.org/10.1159/000330809>

Carels, R. A., Hlavka, R., Selensky, J. C., Solar, C., Rossi, J., & Caroline Miller, J. (2019). A daily diary study of internalised weight bias and its psychological, eating and exercise correlates. *Psychology & Health*, *34*(3), 306–320. <https://doi.org/10.1080/08870446.2018.1525491>

Carels, R. A., Miller, J. C., Hlavka, R., Selensky, J., Shonrock, A. M. T., & Ellis, J. M. (2020). Associations between husbands’ weight bias and related concerns and husbands’ and wives’ psychological and relationship outcomes. *Body Image*, *35*, 11–21. <https://doi.org/10.1016/j.bodyim.2020.07.008>

Carels, R. A., Wott, C. B., Young, K. M., Gumble, A., Koball, A., & Oehlhof, M. W. (2010). Implicit, explicit, and internalized weight bias and psychosocial maladjustment among treatment-seeking adults. *Eating Behaviors*, *11*(3), 180–185. <https://doi.org/10.1016/j.eatbeh.2010.03.002>

Carels, R. A., Young, K. M., Wott, C. B., Harper, J., Gumble, A., Hobbs, M. W., & Clayton, A. M. (2009). Internalized weight stigma and its ideological correlates among weight loss treatment seeking adults. *Eating and Weight Disorders*, *14*(2), 92-97. <https://doi.org/10.1007/BF03327805>

Cassin, S. E., Buchman, D. Z., Leung, S. E., Kantarovich, K., Hawa, A., Carter, A., & Sockalingam, S. (2019). Ethical, stigma, and policy implications of food addiction: A scoping review. *Nutrients*, *11*(4). <https://doi.org/10.3390/nu11040710>

Celik, G., Yilmaz, E., Nazik, F., & Unver, H. (2023). Determination of distress, emotional eating and internalized weight bias levels of Turkish pregnant women. *Journal of Obstetrics and Gynaecology*, *43*(1), 2153020. <https://doi.org/10.1080/01443615.2022.2153020>

Chan, K. L., Lee, C. S. C., Cheng, C. M., Hui, L. Y., So, W. T., Yu, T. S., & Lin, C. Y. (2019). Investigating the relationship between weight-related self-stigma and mental health for overweight/obese children in Hong Kong. *The Journal of Nervous and Mental Disease*, *207*(8), 637–641. <https://doi.org/10.1097/NMD.0000000000001021>

Chao, G. F., Diaz, A., Ghaferi, A. A., Dimick, J. B., & Byrnes, M. E. (2022). Understanding racially diverse community member views of obesity stigma and bariatric surgery. *Obesity Surgery*, *32*(4), 1216–1226. <https://doi.org/10.1007/s11695-022-05928-x>

Chen, H., & Ye, Y. (2021). Validation of the Weight Bias Internalization Scale for mainland Chinese children and adolescents. *Frontiers in Psychology*, *11*. <https://doi.org/10.3389/fpsyg.2020.594949>

Chen, H., Ye, Y., & Guo, J. (2020). Impact of weight stigma on preadolescents’ and adolescents’ disordered eating behaviors: Testing two mediation models. *Social Behavior and Personality*, *48*(10). <https://doi.org/10.2224/sbp.9392>

Chen, I.-H., Huang, P.-C., Lin, Y.-C., Gan, W. Y., Fan, C.-W., Yang, W.-C., Tung, S. E. H., Poon, W. C., Griffiths, M. D., & Lin, C.-Y. (2022). The Yale Food Addiction Scale 2.0 and the modified Yale Food Addiction Scale 2.0 in Taiwan: Factor structure and concurrent validity. *Frontiers in Psychiatry*, *13*, 1014447. <https://doi.org/10.3389/fpsyt.2022.1014447>

Cheng, O. Y., Yam, C. L. Y., Cheung, N. S., Lee, P. L. P., Ngai, M. C., & Lin, C. Y. (2019). Extended theory of planned behavior on eating and physical activity. *American Journal of Health Behavior*, *43*(3), 569–581. <https://doi.org/10.5993/AJHB.43.3.11>

Chirawat, P., Kamolthip, R., Rattaprach, R., Nadhiroh, S. R., Tung, S. E. H., Gan, W. Y., Pinyo, M., Nabpran, T., Rozzell-Voss, K. N., Latner, J. D., & Lin, C.-Y. (2022). Weight stigma among young adults in Thailand: Reliability, validation, and measurement invariance of the Thai-translated Weight Self Stigma Questionnaire and Perceived Weight Stigma Scale. *International Journal of Environmental Research and Public Health*, *19*(23), 15868. <https://doi.org/10.3390/ijerph192315868>

Christensen, B. J., Schmidt, J. B., Nielsen, M. S., Tækker, L., Holm, L., Lunn, S., Bredie, W. L. P., Ritz, C., Holst, J. J., Hansen, T., Hilbert, A., le Roux, C. W., Hulme, O. J., Siebner, H., Morville, T., Naver, L., Floyd, A. K., & Sjödin, A. (2018). Patient profiling for success after weight loss surgery (GO Bypass study): An interdisciplinary study protocol. *Contemporary Clinical Trials Communications*, *10*, 121–130. <https://doi.org/10.1016/j.conctc.2018.02.002>

Christensen, K. A., Forbush, K. T., Cushing, C. C., Lejuez, C. W., Fleming, K. K., & Swinburne Romine, R. E. (2021). Evaluating associations between fitspiration and thinspiration content on Instagram and disordered-eating behaviors using ecological momentary assessment: A registered report. *International Journal of Eating Disorders*, *54*(7), 1307–1315. <https://doi.org/10.1002/eat.23518>

Christian, C., Nicholas, J. K., Penwell, T. E., & Levinson, C. A. (2023). Profiles of experienced and internalized weight-based stigma in college students across the weight spectrum: Associations with eating disorder, depression, and anxiety symptoms. *Eating Behaviors*, *50*, 101772. <https://doi.org/10.1016/j.eatbeh.2023.101772>

Chugh, M., Friedman, A. M., Clemow, L. P., & Ferrante, J. M. (2013). Women weigh in: Obese African American and White women’s perspectives on physicians’ roles in weight management. *The Journal of the American Board of Family Medicine*, *26*(4), 421–428. <https://doi.org/10.3122/jabfm.2013.04.120350>

Ciupitu-Plath, C., Wiegand, S., & Babitsch, B. (2018). The weight bias internalization scale for youth: Validation of a specific tool for assessing internalized weight bias among treatment-seeking German adolescents with overweight. *Journal of Pediatric Psychology*, *43*(1), 40–51. <https://doi.org/10.1093/jpepsy/jsx079>

Constant, A., Moirand, R., Thibault, R., & Val-Laillet, D. (2020). Meeting of minds around food addiction: Insights from addiction medicine, nutrition, psychology, and neurosciences. *Nutrients*, *12*(11). <https://doi.org/10.3390/nu12113564>

Crane, N., Hagerman, C., Horgan, O., & Butryn, M. (2023). Patterns and predictors of engagement with digital self-monitoring during the maintenance phase of a behavioral weight loss program: Quantitative study. *JMIR mHealth and uHealth*, *11*, e45057. <https://doi.org/10.2196/45057>

Craven, M. P., & Fekete, E. M. (2022). Internalized weight stigma, psychological well-being, and sleep in women. *International Journal of Behavioral Medicine*, *29*(2), 199–208. <https://doi.org/10.1007/s12529-021-10008-y>

Crowley, N. (2023). Person-first treatment strategies: Weight bias and impact on mental health of people living with obesity. *Primary Care: Clinics in Office Practice*, *50*(1), 89–101. <https://doi.org/10.1016/j.pop.2022.10.002>

Cuauro, S. E., Santos, N., Andrade, E., Dani, A. W., Sanchious, S. N., Hooper, S. C., & Becker, C. B. (2023). Internalized weight stigma and weight discrimination: Associations with quality of life and psychosocial impairment in a sample living with food insecurity. *International Journal of Environmental Research and Public Health*, *20*(24), 7147. <https://doi.org/10.3390/ijerph20247147>

Curll, S. L., & Brown, P. M. (2020). Weight stigma and psychological distress: A moderated mediation model of social identification and internalised bias. *Body Image*, *35*, 207–216. <https://doi.org/10.1016/j.bodyim.2020.09.006>

Davidsen, E., Pico, M., Sandoe, P., & Lund, T. (2023). “I am very critical of my body, but I am not a worthless person”: A qualitative investigation of internalized weight stigma in Denmark. *Frontiers in Psychology*, *13*. <https://doi.org/10.3389/fpsyg.2022.1049568>

Davies, A. E., Burnette, C. B., Ravyts, S. G., & Mazzeo, S. E. (2022). A randomized control trial of Expand Your Horizon: An intervention for women with weight bias internalization. *Body Image*, *40*, 138–145. <https://doi.org/10.1016/j.bodyim.2021.12.006>

Davison, K. K., Schmalz, D. L., Young, L. M., & Birch, L. L. (2008). Overweight girls who internalize fat stereotypes report low psychosocial well‐being. *Obesity*, *16*(S2), S30-S38. <https://doi.org/10.1038/oby.2008.451>

Decker, K. M., Thurston, I. B., & Kamody, R. C. (2018). The mediating role of internalized weight stigma on weight perception and depression among emerging adults: Exploring moderation by weight and race. *Body Image*, *27*, 202–210. <https://doi.org/10.1016/j.bodyim.2018.10.004>

Dieterich, R., Chang, J., Danford, C., Scott, P. W., Wend C., & Demirci, J. (2021). The relationship between internalized weight stigma during pregnancy and breastfeeding: A prospective longitudinal study. *Obesity*, *29*(5), 919–927. <https://doi.org/10.1002/oby.23139>

Dilsiz, N. B., & Arslan, S. (2023). Investigation of the relationship between weight self-stigma, emotional eating, and diet satisfaction in obese individuals. *European Research Journal*, *9*(2), 407–415. <https://doi.org/10.18621/eurj.1250216>

Dochat, C., Afari, N., Wooldridge, J. S., Herbert, M. S., Gasperi, M., & Lillis, J. (2020). Confirmatory factor analysis of the Acceptance and Action Questionnaire for Weight-Related Difficulties-Revised (AAQW-R) in a United States sample of adults with overweight and obesity. *Journal of Contextual Behavioral Science*, *15*, 189–196. <https://doi.org/10.1016/j.jcbs.2020.01.006>

Donahue, M. L., Levin, M. E., Olson, K., Panza, E., & Lillis, J. (2023). Examining the role of experiential avoidance and valued action in the negative effects of weight self-stigma. *Journal of Behavioral Medicine*, *46*(3), 517–524. <https://doi.org/10.1007/s10865-022-00378-3>

Douglas, V., & Varnado-Sullivan, P. (2016). Weight stigmatization, internalization, and eating disorder symptoms: The role of emotion dysregulation. *Stigma and Health*, *1*(3), 166–175. <https://doi.org/10.1037/sah0000029>

Dunaev, J., Markey, C. H., & Brochu, P. M. (2018). An attitude of gratitude: The effects of body-focused gratitude on weight bias internalization and body image. *Body Image*, *25*, 9–13. <https://doi.org/10.1016/j.bodyim.2018.01.006>

Durso, L. E., & Latner, J. D. (2008). Understanding self‐directed stigma: Development of the weight bias internalization scale. *Obesity*, *16*(S2), S80-S86. <https://doi.org/10.1038/oby.2008.448>

Durso, L. E., Latner, J. D., & Ciao, A. C. (2016). Weight bias internalization in treatment-seeking overweight adults: Psychometric validation and associations with self-esteem, body image, and mood symptoms. *Eating Behaviors*, *21*, 104–108. <https://doi.org/10.1016/j.eatbeh.2016.01.011>

Durso, L. E., Latner, J. D., & Hayashi, K. (2012). Perceived discrimination is associated with binge eating in a community sample of non-overweight, overweight, and obese adults. *Obesity Facts*, *5*(6), 869–880. <https://doi.org/10.1159/000345931>

Durso, L. E., Latner, J. D., White, M. A., Masheb, R. M., Blomquist, K. K., Morgan, P. T., & Grilo, C. M. (2012). Internalized weight bias in obese patients with binge eating disorder: Associations with eating disturbances and psychological functioning. *International Journal of Eating Disorders*, *45*(3), 423–427. <https://doi.org/10.1002/eat.20933>

Eisenberg, M. H., Street, R. L. Jr., & Persky, S. (2017). “It runs in my family …”: The association of perceived family history with body dissatisfaction and weight bias internalization among overweight women. *Women & Health*, 57(4), 478–493. <https://doi.org/10.1080/03630242.2016.1170095>

Endo, S., Kasuga, H., Yusuke, M., Hidaka, T., Kakamu, T., & Fukushima, T. (2022). Reliability and validity of the Japanese version of the weight bias internalization scale. *BMC* *Research Notes*, *15*(1), 333. <https://doi.org/10.1186/s13104-022-06221-x>

English, S., & Vallis, M. (2023). Moving beyond eat less, move more using willpower: Reframing obesity as a chronic disease impact of the 2020 Canadian obesity guidelines reframed narrative on perceptions of self and the patient-provider relationship. *Clinical Obesity*, *13*(6), e12615. <https://doi.org/10.1111/cob.12615>

Erdogan, Z., Kurcer, M. K., Kurtuncu, M., & Catalcam, S. (2018). Validity and reliability of the Turkish version of the weight Self-stigma questionnaire. *The Journal of the Pakistan Medical Association*, *68*(12), 1798–1803.

Essayli, J. H., Murakami, J. M., Wilson, R. E., & Latner, J. D. (2017). The impact of weight labels on body image, internalized weight stigma, affect, perceived health, and intended weight loss behaviors in normal-weight and overweight college women. *American Journal of Health Promotion*, *31*(6), 484–490. <https://doi.org/10.1177/0890117116661982>

Fan, C.-W., Chang, Y.-L., Huang, P.-C., Fung, X. C. C., Chen, J.-K., Bevan, N., O’Brien, K.S., Yeh, Y.-C., Chen, H.-P., Chen, I.-H., Lin, I.-C., Griffiths, M. D., & Lin, C.-Y. (2023). The tendency to avoid physical activity and sport scale (TAPAS): Rasch analysis with differential item functioning testing among a Chinese sample. *BMC Psychology*, *11*(1), 369. <https://doi.org/10.1186/s40359-023-01377-y>

Fan, C.-W., Huang, P.-C., Chen, I.-H., Huang, Y.-T., Chen, J.-S., Fung, X. C. C., Chen, J.-K., Yang, Y.-N., O’Brien, K. S., Lin, C.-Y., & Griffiths, M. D. (2023). Differential item functioning for the tendency of avoiding physical activity and sport scale across two subculture samples: Taiwanese and mainland Chinese university students. *Heliyon*, *9*(12), e22583. <https://doi.org/10.1016/j.heliyon.2023.e22583>

Fan, C. W., Liu, C. H., Huang, H. H., Lin, C. Y., & Pakpour, A. H. (2021). Weight stigma model on quality of life among children in Hong Kong: A cross-sectional modeling study. *Frontiers in Psychology*, *12*, 629786. <https://doi.org/10.3389/fpsyg.2021.629786>

Farhangi, M. A., Emam-Alizadeh, M., Hamedi, F., & Jahangiry, L. (2017). Weight self-stigma and its association with quality of life and psychological distress among overweight and obese women. *Eating and Weight Disorders*, *22*(3), 451–456. <https://doi.org/10.1007/s40519-016-0288-2>

Feig, E. H., Amonoo, H. L., Onyeaka, H. K., Romero, P. M., Kim, S., & Huffman, J. C. (2020). Weight bias internalization and its association with health behaviour adherence after bariatric surgery. *Clinical Obesity*, *10*(4), e12361. <https://doi.org/10.1111/cob.12361>

Fekete, E., Herndier, R., & Sander, A. (2021). Self-compassion, internalized weight stigma, psychological well-being, and eating behaviors in women. *Mindfulness*, *12*(5), 1262–1271. <https://doi.org/10.1007/s12671-021-01597-6>

Fekih-Romdhane, F., He, J., Malaeb, D., Dabbous, M., Hallit, R., Obeid, S., & Hallit, S. (2023). Psychometric properties of the Arabic versions of the Three-Item Short Form of the modified Weight Bias Internalization Scale (WBIS-3) and the Muscularity Bias Internalization Scale (MBIS). *Journal of Eating Disorders*, *11*(1), 82. <https://doi.org/10.1186/s40337-023-00805-z>

Fields, L. C., Brown, C., Skelton, J. A., Cain, K. S., & Cohen, G. M. (2021). Internalized weight bias, teasing, and self-esteem in children with overweight or obesity. *Childhood Obesity*, *17*(1), 43–50. <https://doi.org/10.1089/chi.2020.0150>

Flint, S. W., Raisborough, J., & Hudson, J. (2019). Editorial: The implications of weight bias internalization. *Frontiers in Psychology*, *10*, 3019. <https://doi.org/10.3389/fpsyg.2019.03019>

Forbes, Y., & Donovan, C. (2019). The role of internalised weight stigma and self-compassion in the psychological well-being of overweight and obese women. *Australian Psychologist*, *54*(6), 471–482. <https://doi.org/10.1111/ap.12407>

Forbes, Y. N., Moffitt, R. L., Van Bokkel, M., & Donovan, C. L. (2020). Unburdening the weight of stigma: Findings from a compassion-focused group program for women with overweight and obesity. *Journal of Cognitive Psychotherapy*, *34*(4), 336–357. <https://doi.org/10.1891/JCPSY-D-20-00015>

Forouhar, V., Edache, I. Y., Salas, X. R., & Alberga, A. S. (2023). Weight bias internalization and beliefs about the causes of obesity among the Canadian public. *BMC Public Health*, *23*(1), 1621. <https://doi.org/10.1186/s12889-023-16454-5>

Fung, X. C. C., Pakpour, A. H., Wu, Y. K., Fan, C. W., Lin, C. Y., & Tsang, H. W. H. (2019). Psychosocial variables related to weight-related self-stigma in physical activity among young adults across weight status. *International Journal of Environmental Research and Public Health*, *17*(1). <https://doi.org/10.3390/ijerph17010064>

Gailey, J., & Harjunen, H. (2019). A cross-cultural examination of fat women’s experiences: Stigma and gender in North American and Finnish culture. *Feminism & Psychology*, *29*(3), 374–390. <https://doi.org/10.1177/0959353518819582>

Gan, W. Y., Tung, S. E. H., Kamolthip, R., Ghavifekr, S., Chirawat, P., Nurmala, I., Chang, Y.-L., Latner, J. D., Huang, R.-Y., & Lin, C.-Y. (2022). Evaluation of two weight stigma scales in Malaysian university students: Weight self-stigma questionnaire and perceived weight stigma scale. *Eating and Weight Disorders*, *27*(7), 2595–2604. <https://doi.org/10.1007/s40519-022-01398-3>

Gaston-Panthaki, A., Serrano, A., Virani, N., Sylvestre, J., Crisafulli, B. F., & Becker, C. B. (2023). Food insecurity, weight-based discrimination, weight self-stigma, and mental health in post-bariatric surgery patients. *Body Image*, *45*, 46–53. <https://doi.org/10.1016/j.bodyim.2023.01.009>

Gerend, M. A., Stewart, C., & Wetzel, K. (2022). Vulnerability and resilience to the harmful health consequences of weight discrimination in Black, Latina, and sexual minority women. *Social Science and Medicine*, *315*, 115555. <https://doi.org/10.1016/j.socscimed.2022.115555>

Gmeiner, M. S., & Warschburger, P. (2020). Intrapersonal predictors of weight bias internalization among elementary school children: A prospective analysis. *BMC Pediatrics*, *20*(1), 408. <https://doi.org/10.1186/s12887-020-02264-w>

Gmeiner, M. S., & Warschburger, P. (2022). Simply too much: The extent to which weight bias internalization results in a higher risk of eating disorders and psychosocial problems. *Eating and Weight Disorders*, *27*(1), 317–324. <https://doi.org/10.1007/s40519-021-01170-z>

Godoy-Izquierdo, D., González-Hernández, J., Rodríguez-Tadeo, A., Lara, R., Ogallar, A., Navarrón, E., Ramírez, M. J., López-Mora, C., & Arbinaga, F. (2020). Body satisfaction, weight stigma, positivity, and happiness among Spanish adults with overweight and obesity. *International Journal of Environmental Research and Public Health*, *17*(12). <https://doi.org/10.3390/ijerph17124186>

Godoy-Izquierdo, D., Lara, R., Ogallar, A., Rodríguez-Tadeo, A., Ramírez, M. J., Navarrón, E., & Arbinaga, F. (2021). Psychosocial and diet-related lifestyle clusters in overweight and obesity. *International Journal of Environmental Research and Public Health*, *18*(12). <https://doi.org/10.3390/ijerph18126461>

Griffiths, C., Williamson, H., Zucchelli, F., Paraskeva, N., & Moss, T. (2018). A systematic review of the effectiveness of acceptance and commitment therapy (ACT) for body image dissatisfaction and weight self-stigma in adults. *Journal of Contemporary Psychotherapy*, *48*(4), 189–204. <https://doi.org/10.1007/s10879-018-9384-0>

Groshon, L. C., & Pearl, R. L. (2023). Longitudinal associations of binge eating with internalized weight stigma and eating self-efficacy. *Eating Behaviors*, *50*, 101785. <https://doi.org/10.1016/j.eatbeh.2023.101785>

Hain, B., Langer, L., Hünnemeyer, K., Rudofsky, G., Zech, U., & Wild, B. (2015). Translation and validation of the German version of the weight self-stigma questionnaire (WSSQ). *Obesity Surgery*, *25*(4), 750–753. <https://doi.org/10.1007/s11695-015-1598-6>

Haley, E. N., Dolbier, C. L., Carels, R. A., & Whited, M. C. (2022). A brief pilot self-compassion intervention for women with overweight/obesity and internalized weight bias: Feasibility, acceptability, and future directions. *Journal of Contextual Behavioral Science*, *23*, 59–63. <https://doi.org/10.1016/j.jcbs.2021.12.001>

Han, S., Agostini, G., Brewis, A. A., & Wutich, A. (2018). Avoiding exercise mediates the effects of internalized and experienced weight stigma on physical activity in the years following bariatric surgery. *BMC Obesity*, *5*, 18. <https://doi.org/10.1186/s40608-018-0195-3>

Hayward, L. E., Vartanian, L. R., & Pinkus, R. T. (2018). Weight stigma predicts poorer psychological well-being through internalized weight bias and maladaptive coping responses. *Obesity*, *26*(4), 755–761. <https://doi.org/10.1002/oby.22126>

Hilbert, A., Baldofski, S., Zenger, M., Löwe, B., Kersting, A., & Braehler, E. (2014). Weight bias internalization scale: Psychometric properties and population norms. *PLoS One*, *9*(1), e86303. <https://doi.org/10.1371/journal.pone.0086303>

Hilbert, A., Braehler, E., Haeuser, W., & Zenger, M. (2014). Weight bias internalization, core self-evaluation, and health in overweight and obese persons. *Obesity*, *22*(1), 79–85. <https://doi.org/10.1002/oby.20561>

Hilbert, A., Braehler, E., Schmidt, R., Löwe, B., Häuser, W., & Zenger, M. (2015). Self-compassion as a resource in the self-stigma process of overweight and obese individuals. *Obesity Facts*, *8*(5), 293–301. <https://doi.org/10.1159/000438681>

Hilbert, A., Hübner, C., Schmutzer, G., Danielsdottir, S., Brähler, E., & Puhl, R. (2017). Public support for weight-related antidiscrimination laws and policies. *Obesity Facts*, *10*(2), 101–111. <https://doi.org/10.1159/000456012>

Himmelstein, M. S., & Puhl, R. M. (2019). Weight-based victimization from friends and family: Implications for how adolescents cope with weight stigma. *Pediatric Obesity*, *14*(1). <https://doi.org/10.1111/ijpo.12453>

Himmelstein, M. S., & Puhl, R. M. (2021). At multiple fronts: Diabetes stigma and weight stigma in adults with type 2 diabetes. *Diabetic Medicine*, *38*(1), e14387. <https://doi.org/10.1111/dme.14387>

Himmelstein, M. S., Puhl, R. M., Pearl, R. L., Pinto, A. M., & Foster, G. D. (2020). Coping with weight stigma among adults in a commercial weight management sample. *International Journal of Behavioral Medicine*, *27*(5), 576–590. <https://doi.org/10.1007/s12529-020-09895-4>

Himmelstein, M. S., Puhl, R. M., & Quinn, D. M. (2017). Intersectionality: An understudied framework for addressing weight stigma. *American Journal of Preventative Medicine*, *53*(4), 421–431. <https://doi.org/10.1016/j.amepre.2017.04.003>

Himmelstein, M. S., Puhl, R. M., & Quinn, D. M. (2019). Overlooked and understudied: Health consequences of weight stigma in men. *Obesity*, *27*(10), 1598–1605. <https://doi.org/10.1002/oby.22599>

Hopkins, C. M., & Bennett, G. G. (2018). Weight-related terms differentially affect self-efficacy and perception of obesity. *Obesity*, *26*(9), 1405–1411. <https://doi.org/10.1002/oby.22255>

Hopkins, C. M, Miller, H. N., Brooks, T. L., Mo-Hunter, L., Steinberg, D. M., & Bennett, G. G. (2021). Designing ruby: Protocol for a 2-arm, brief, digital randomized controlled trial for internalized weight bias. *JMIR Research Protocols*, *10*(11), e31307. <https://doi.org/10.2196/31307>

Horn, F., & Jongenelis, M. I. (2022). Outcomes of exposure to healthy weight and lifestyle advertising: An experimental study of adults from the United Kingdom. *Preventative Medicine Reports*, *25*, 101679. <https://doi.org/10.1016/j.pmedr.2021.101679>

Huang, P.-C., Lee, C.-H., Griffiths, M. D., O’Brien, K. S., Lin, Y.-C., Gan, W. Y., Poon, W. C., Hung, C.-H., Lee, K.-H., & Lin, C.-Y. (2022). Sequentially mediated effects of weight-related self-stigma and psychological distress in the association between perceived weight stigma and food addiction among Taiwanese university students: A cross-sectional study. *Journal of Eating Disorders*, *10*(1), 177. <https://doi.org/10.1186/s40337-022-00701-y>

Hübner, C., Baldofski, S., Zenger, M., Tigges, W., Herbig, B., Jurowich, C., Kaiser, S., Dietrich, A., & Hilbert, A. (2015). Influences of general self-efficacy and weight bias internalization on physical activity in bariatric surgery candidates. *Surgery for Obesity and Related Diseases*, *11*(6), 1371–1376. <https://doi.org/10.1016/j.soard.2014.11.013>

Hübner, C., Schmidt, R., Selle, J., Köhler, H., Müller, A., de Zwaan, M., & Hilbert, A. (2016). Comparing self-report measures of internalized weight stigma: The Weight Self-Stigma Questionnaire versus the Weight Bias Internalization Scale. *PLoS One*, *11*(10), e0165566. <https://doi.org/10.1371/journal.pone.0165566>

Huellemann, K., & Calogero, R. (2020). Self-compassion and body checking among women: The mediating role of stigmatizing self-perceptions. *Mindfulness*, *11*(9), 2121–2130. <https://doi.org/10.1007/s12671-020-01420-8>

Huellemann, K. L., Tremblay, P. F., & Calogero, R. M. (2023). Effects of online self-compassionate writing on stigmatizing and affirming self-perceptions: Potential boundary conditions in undergraduate women. *Body Image*, *46*, 406–418. <https://doi.org/10.1016/j.bodyim.2023.07.008>

Inderstrodt-Stephens, J., & Acharya, L. (2018). “Fat” chicks who run: Stigma experienced by “overweight” endurance athletes. *Journal of Sport & Social Issues*, *42*(1), 49–67. <https://doi.org/10.1177/0193723517747884>

Innamorati, M., Imperatori, C., Lamis, D., Contardi, A., Castelnuovo, G., Tamburello, S., Manzoni, G., & Fabbricatore, M. (2017). Weight Bias Internalization Scale discriminates obese and overweight patients with different severity levels of depression: The Italian version of the WBIS. *Current Psychology*, *36*(2), 242–251. <https://doi.org/10.1007/s12144-016-9406-6>

Iturbe, I., Pereda-Pereda, E., Echeburúa, E., & Maiz, E. (2021). The effectiveness of an acceptance and commitment therapy and mindfulness group intervention for enhancing the psychological and physical well-being of adults with overweight or obesity seeking treatment: The Mind & Life randomized control trial study protocol. *International Journal of Environmental Research and Public Health*, *18*(9). <https://doi.org/10.3390/ijerph18094396>

Jackson, A., Busig, J., & Lanigan, J. (2022). “Thin and muscular”: A cross-sectional mixed methods study of people’s descriptions of health. *Stigma and Health*, *7*(4), 389–395. <https://doi.org/10.1037/sah0000411>

Jackson, A. M., Cox, A. E., Sano, Y., Parker, L., & Lanigan, J. (2022). Body image and eating behaviors: A latent profile analysis. *Body Image*, *41*, 396–405. <https://doi.org/10.1016/j.bodyim.2022.04.013>

Jimenez-Loaisa, A., Beltran-Carrillo, V., Gonzalez-Cutre, D., & Jennings, G. (2020). Healthism and the experiences of social, healthcare and self-stigma of women with higher-weight. *Social Theory & Health*, *18*(4), 410–424. <https://doi.org/10.1057/s41285-019-00118-9>

Jiwanmall, S. A., Kattula, D., Nandyal, M. B., Parvathareddy, S., Kirubakaran, R., Jebasingh, F., Paul, T. V., Thomas, N., & Kapoor, N. (2022). Weight stigma in patients with obesity and its clinical correlates: A perspective from an Indian bariatric clinic. *Cureus*, *14*(7), e26837. <https://doi.org/10.7759/cureus.26837>

Jones, J. M., Schönherr, D. M., Zaitsoff, S., & Pullmer, R. (2019). Changing from the inside out? Examining relationships between overweight identification, dieting behaviours, and body measurements over time. *British Journal of Health Psychology*, *24*(2), 460–476. <https://doi.org/10.1111/bjhp.12363>

Jongenelis, M., Dixon, H., Scully, M., & Morley, B. (2023). Exploring intended and unintended reactions to healthy weight and lifestyle advertisements: An online experiment. *Health Education & Behavior*, *50*(1), 58–69. <https://doi.org/10.1177/10901981221104727>

Jung, F., Spahlholz, J., Hilbert, A., Riedel-Heller, S. G., & Luck-Sikorski, C. (2017). Impact of weight-related discrimination, body dissatisfaction and self-stigma on the desire to weigh less. *Obesity Facts*, *10*(2), 139–151. <https://doi.org/10.1159/000468154>

Jung, F. U., Bae, Y. J., Kratzsch, J., Riedel-Heller, S. G., & Luck-Sikorski, C. (2020). Internalized weight bias and cortisol reactivity to social stress. *Cognitive, Affective, & Behavioral Neuroscience*, *20*(1), 49–58. <https://doi.org/10.3758/s13415-019-00750-y>

Jung, F. U., & Luck-Sikorski, C. (2019). Overweight and lonely? A representative study on loneliness in obese people and its determinants. *Obesity Facts*, *12*(4), 440–447. <https://doi.org/10.1159/000500095>

Keast, R., Withnell, S., & Bodell, L. P. (2023). Longitudinal associations between weight stigma and disordered eating across the weight spectrum. *Eating Behaviors*, *50*, 101788. <https://doi.org/10.1016/j.eatbeh.2023.101788>

Keirns, N. G., Keirns, B. H., Tsotsoros, C. E., Sciarrillo, C. M., Emerson, S. R., & Hawkins, M. A. W. (2022). Associations between internalized weight stigma and visceral adipose tissue status are observed in women but not men. *Stigma and Health*, *7*(2), 161–168. <https://doi.org/10.1037/sah0000381>

Keirns, N. G., Tsotsoros, C. E., Addante, S., Layman, H. M., Krems, J. A., Pearl, R. L., Tomiyama, J. A., & Hawkins M. A. W. (2021). Adverse childhood experiences associated with greater internalization of weight stigma in women with excess weight. *Obesities*, *1*(1), 49–57. <https://doi.org/10.3390/obesities1010005>

Khodari, B. H., Shami, M. O., Shajry, R. M., Shami, J. A., Names, A. A., Alamer, A. A., Moafa, A. M., Hakami, R. O., Almuhaysin, G. S., & Alqassim, A. Y. (2021). The relationship between weight self-stigma and quality of life among youth in the Jazan region, Saudi Arabia. *Cureus*, *13*(9), e18158. <https://doi.org/10.7759/cureus.18158>

Kliem, S., Puls, H. C., Hinz, A., Kersting, A., Brähler, E., & Hilbert, A. (2020). Validation of a three-item short form of the Modified Weight Bias Internalization Scale (WBIS-3) in the German population. *Obesity Facts*, *13*(6), 560–571. <https://doi.org/10.1159/000510923>

Kline, K. M., O’Neill, E. A., Behar, S., Winter, V. R., & Clemens, J. P. (2023). Weight stigma: A potential barrier to psychiatric/mental health medication care. *Social Work in Mental Health*, *21*(5), 492–509. <https://doi.org/10.1080/15332985.2023.2184191>

Koball, A. M., Mueller, P. S., Craner, J., Clark, M. M., Nanda, S., Kebede, E. B., & Grothe, K. B. (2018). Crucial conversations about weight management with healthcare providers: Patients’ perspectives and experiences. *Eating and Weight Disorders*, *23*(1), 87–94. <https://doi.org/10.1007/s40519-016-0304-6>

Lacroix, E., Alberga, A., Russell-Mathew, S., McLaren, L., & Von Ranson, K. (2017). Weight bias: A systematic review of characteristics and psychometric properties of self-report questionnaires. *Obesity Facts*, *10*(3), 223–237. <https://doi.org/10.1159/000475716>

Latner, J. D., Barile, J. P., Durso, L. E., & O’Brien, K. S. (2014). Weight and health-related quality of life: The moderating role of weight discrimination and internalized weight bias. *Eating Behaviors*, *15*(4), 586–590. <https://doi.org/10.1016/j.eatbeh.2014.08.014>

Latner, J. D., Durso, L. E., & Mond, J. M. (2013). Health and health-related quality of life among treatment-seeking overweight and obese adults: Associations with internalized weight bias. *Journal of Eating Disorders*, *1*, 3. <https://doi.org/10.1186/2050-2974-1-3>

Lawrence, S. E., Puhl, R. M., Watson, R. J., Schwartz, M. B., Lessard, L. M., & Foster, G. D. (2023). Family-based weight stigma and psychosocial health: A multinational comparison. *Obesity*, *31*(6), 1666–1677. <https://doi.org/10.1002/oby.23748>

Lawson, J. L., LeCates, A., Ivezaj, V., Lydecker, J., & Grilo, C. M. (2020). Internalized weight bias and loss-of-control eating following bariatric surgery. *Eating Disorders*, *29*(6), 630-643. <https://doi.org/10.1080/10640266.2020.1731920>

Lawson, J. L., Schuh, L. M., Creel, D. B., Blackinton, R. M., Giambrone, S. A., Grilo, C. M., & Ivezaj, V. (2021). Examining weight bias and loss-of-control eating among individuals seeking bariatric surgery. *Obesity Surgery*, *31*(8), 3498–3505. <https://doi.org/10.1007/s11695-021-05418-6>

Lee, M. S., & Dedrick, R. F. (2016). Weight Bias Internalization Scale: Psychometric properties using alternative weight status classification approaches. *Body Image*, *17*, 25–29. <https://doi.org/10.1016/j.bodyim.2016.01.008>

Lee, M. S., Gonzalez, B. D., Small, B. J., & Thompson, J. K. (2019). Internalized weight bias and psychological wellbeing: An exploratory investigation of a preliminary model. *PLoS One*, *14*(5), e0216324. <https://doi.org/10.1371/journal.pone.0216324>

Leget, D. L., LaCaille, L. J., & Pearl, R. L. (2023). Comparing measurement of internalized weight stigma and body dissatisfaction. *Body Image*, *47*, 101622. <https://doi.org/10.1016/j.bodyim.2023.101622>

Lent, M. R., Napolitano, M. A., Wood, G. C., Argyropoulos, G., Gerhard, G. S., Hayes, S., Foster, G. D., Collins, C. A., & Still, C. D. (2014). Internalized weight bias in weight-loss surgery patients: Psychosocial correlates and weight loss outcomes. *Obesity Surgery*, *24*(12), 2195–2199. <https://doi.org/10.1007/s11695-014-1455-z>

Lessard, L. M., Puhl, R. M., Foster, G. D., & Cardel, M. I. (2023). Parental communication about body weight and adolescent health: The role of positive and negative weight-related comments. *Journal of Pediatric Psychology*, *48*(8), 700–706. <https://doi.org/10.1093/jpepsy/jsad040>

Levin, M., Potts, S., Haeger, J., & Lillis, J. (2018). Delivering acceptance and commitment therapy for weight self-stigma through guided self-help: results from an open pilot trial. *Cognitive and Behavioral Practice*, *25*(1), 87–104.

Levin, M. E., Petersen, J. M., Durward, C., Bingeman, B., Davis, E., Nelson, C., & Cromwell, S. (2021). A randomized controlled trial of online acceptance and commitment therapy to improve diet and physical activity among adults who are overweight/obese. *Translational Behavioral Medicine*, *11*(6), 1216–1225. <https://doi.org/10.1093/tbm/ibaa123>

Levy, M., Forouhar, V., Edache, I. Y., & Alberga, A. S. (2023). Predictors of support for anti-weight discrimination policies among Canadian adults. *Frontiers in Public Health*, *11*, 1060794. <https://doi.org/10.3389/fpubh.2023.1060794>

Levy, M., Kakinami, L., & Alberga, A. S. (2022). The relationship between weight bias internalization and healthy and unhealthy weight control behaviours. *Eating and Weight Disorders*, *27*, 1621-1632. <https://doi.org/10.1007/s40519-021-01291-5>

Levy, M., Nguyen, A., Kakinami, L., & Alberga, A. S. (2023). Weight bias internalization: Relationships with mental health, physical activity, and sedentary behavior. *Stigma and Health*, *8*(4), 453–461. <https://doi.org/10.1037/sah0000336>

Lillis, J., Levin, M. E., & Hayes, S. C. (2011). Exploring the relationship between body mass index and health-related quality of life: A pilot study of the impact of weight self-stigma and experiential avoidance. *Journal of Health Psychology*, *16*(5), 722–727. <https://doi.org/10.1177/1359105310388321>

Lillis, J., Luoma, J. B., Levin, M. E., & Hayes, S. C. (2010). Measuring weight self‐stigma: The weight self‐stigma questionnaire. *Obesity*, *18*(5), 971–976. <https://doi.org/10.1038/oby.2009.353>

Lillis, J., Thomas, J. G, Levin, M. E., & Wing, R. R. (2020). Self-stigma and weight loss: The impact of fear of being stigmatized. *Journal of Health Psychology*, *25*(7), 922–930. <https://doi.org/10.1177/1359105317739101>

Lillis, J., Thomas, J. G., Olson, K., & Wing, R. R. (2019). Weight self-stigma and weight loss during behavioural weight loss intervention. *Obesity Science and Practice*, *5*(1), 21–27. <https://doi.org/10.1002/osp4.314>

Lin, C. Y., Imani, V., Broström, A., Huus, K., Björk, M., Hodges, E. A., & Pakpour, A. H. (2020). Psychological distress and quality of life in Iranian adolescents with overweight/obesity: Mediating roles of weight bias internalization and insomnia. *Eating and Weight Disorders*, *25*(6), 1583–1592. <https://doi.org/10.1007/s40519-019-00795-5>

Lin, C. Y., Imani, V., Cheung, P., & Pakpour, A. H. (2020). Psychometric testing on two weight stigma instruments in Iran: Weight Self-Stigma Questionnaire and Weight Bias Internalized Scale. *Eating and Weight Disorders*, *25*(4), 889–901. <https://doi.org/10.1007/s40519-019-00699-4>

Lin, C. Y., Tsai, M. C., Liu, C. H., Lin, Y. C., Hsieh, Y. P., & Strong, C. (2019). Psychological pathway from obesity-related stigma to depression via internalized stigma and self-esteem among adolescents in Taiwan. *International Journal of Environmental Research and Public Health*, *16*(22). <https://doi.org/10.3390/ijerph16224410>

Lin, K. P., & Lee, M. L. (2017). Validating a Chinese version of the Weight Self‐stigma Questionnaire for use with obese adults. *International Journal of Nursing Practice*, *23*(4), e12537. <https://doi.org/10.1111/ijn.12537>

Lin, S. W., Tsay, S. L., & Lin, K. P. (2021). Prediction factors of weight control intention in Chinese young adults. *International Journal of Nursing Practice*, *27*(3), e12927. <https://doi.org/10.1111/ijn.12927>

Lin, Y.-C., Lin, C.-Y., Saffari, M., Tsai, M.-C., Chang, Y.-H., Strong, C., Chen, J.-K., Hsieh, Y.-P., Yang, Y.-N., & Latner, J. D. (2023). Weight stigma is associated with body mass index among college students in Taiwan: The mediated role of internalized weight stigma. *BMC Psychology*, *11*(1), 365. <https://doi.org/10.1186/s40359-023-01414-w>

Lin, Y. W., Lin, C. Y., Strong, C., Liu, C. H., Hsieh, Y. P., Lin, Y. C., & Tsai, M. C. (2021). Psychological correlates of eating behavior in overweight/obese adolescents in Taiwan: Psychometric and correlation analysis of the Three-Factor Eating Questionnaire (TFEQ)-R21. *Pediatrics & Neonatology*, *62*(1), 41–48. <https://doi.org/10.1016/j.pedneo.2020.08.006>

Lippa, N. C., & Sanderson, S. C. (2013). Impact of informing overweight individuals about the role of genetics in obesity: An online experimental study. *Human Heredity*, *75*(2–4), 186–203. <https://doi.org/10.1159/000353712>

Liu, W., Chen, J.-S., Gan, W. Y., Poon, W. C., Tung, S. E. H., Lee, L. J., Xu, P., Chen, I.-H., Griffiths, M. D., & Lin, C.-Y. (2022). Associations of problematic internet use, weight-related self-stigma, and nomophobia with physical activity: Findings from Mainland China, Taiwan, and Malaysia. *International Journal of Environmental Research and Public Health*, *19*(19), 12135. <https://doi.org/10.3390/ijerph191912135>

Liu, X., Zhang, W., Yue, W., Sun, C., & Li, W. (2022). From weight bias internalization to health-related quality of life: Self-esteem and psychopathology in pre-bariatric surgery patients. *Obesity Surgery*, *32*(11), 3705–3713. <https://doi.org/10.1007/s11695-022-06261-z>

Lozano-Sufrategui, L., Carless, D., Pringle, A., Sparkes, A., & Mckenna, J. (2016). “Sorry mate, you’re probably a bit too fat to be able to do any of these”: Men’s experiences of weight stigma. *International Journal of Men’s Health*, *15*(1), 4–23. <https://doi.org/10.3149/jmh.1501.4>

Lucibello, K. M., Nesbitt, A. E., Solomon-Krakus, S., & Sabiston, C. M. (2021). Internalized weight stigma and the relationship between weight perception and negative body-related self-conscious emotions. *Body Image*, *37*, 84–88. <https://doi.org/10.1016/j.bodyim.2021.01.010>

Lucibello, K. M., Sabiston, C. M., Pila, E., & Arbour-Nicitopoulos, K. (2023). An integrative model of weight stigma, body image, and physical activity in adolescents. *Body Image*, *45*, 1–10. <https://doi.org/10.1016/j.bodyim.2023.01.003>

Macho, S., Andrés, A., & Saldaña, C. (2021). Validation of the modified weight bias internalization scale in a Spanish adult population. *Clinical Obesity*, *11*(4), e12454. <https://doi.org/10.1111/cob.12454>

Macho, S., Andres, A., & Saldana, C. (2023). Weight discrimination, BMI, or weight bias internalization? Testing the best predictor of psychological distress and body dissatisfaction. *Obesity*, *31*(8), 2178–2188. <https://doi.org/10.1002/oby.23802>

Magallares, A., Bolaños-Rios, P., Ruiz-Prieto, I., de Valle, P. B., Irles, J. A., & Jáuregui-Lobera, I. (2017). The mediational effect of weight self-stigma in the relationship between blatant and subtle discrimination and depression and anxiety. *The Spanish Journal of Psychology*, *20*, E4. <https://doi.org/10.1017/sjp.2017.1>

Magallares, A., de Valle, P. B., Irles, J. A., Recio, P., & Jauregui-Lobera, I. (2022). Psychometric properties of the Spanish version of the Weight Self-Stigma Questionnaire (S-WSSQ) in a sample of participants with obesity seeking weight loss treatment. *Eating and Weight Disorders*, *27*(8), 3685–3693. <https://doi.org/10.1007/s40519-022-01511-6>

Mahfoud, D., Fekih-Romdhane, F., Abou Zeid, J., Rustom, L., Mouez, C., Haddad, G., & Hallit, S. (2023). Functionality appreciation is inversely associated with positive psychotic symptoms in overweight/obese patients with schizophrenia. *BMC Psychiatry*, *23*(1), 306. <https://doi.org/10.1186/s12888-023-04795-9>

Maïano, C., Aimé, A., Lepage, G., & Morin, A. J. S. (2019). Psychometric properties of the Weight Self-Stigma Questionnaire (WSSQ) among a sample of overweight/obese French-speaking adolescents. *Eating and Weight Disorders*, *24*(3), 575–583. <https://doi.org/10.1007/s40519-017-0382-0>

Malterud, K., & Ulriksen, K. (2011). Obesity, stigma, and responsibility in health care: A synthesis of qualitative studies. *International Journal of Qualitative Studies on Health and Well-Being*, *6*(4). <https://doi.org/10.3402/qhw.v6i4.8404>

Marshall, R., Latner, J., & Masuda, A. (2020). Internalized weight bias and disordered eating: The mediating role of body image avoidance and drive for thinness. *Frontiers in Psychology*, *10*. <https://doi.org/10.3389/fpsyg.2019.02999>

Martin-Wagar, C. A., Attaway, S. E., & Melcher, K. A. (2023). Differences among feminist and non-feminist women on weight bias internalization, body image, and disordered eating. *Journal of Eating Disorders*, *11*(1), 129. <https://doi.org/10.1186/s40337-023-00851-7>

Martin-Wagar, C. A., & Weigold, I. K. (2023). Internalized stigma as a transdiagnostic factor for women with eating disorders. *Eating Disorders*, *31*(2), 173–190. <https://doi.org/10.1080/10640266.2022.2095481>

McEntee, M. L., Philip, S. R., & Phelan, S. M. (2023). Dismantling weight stigma in eating disorder treatment: Next steps for the field. *Frontiers in Psychiatry*, *14*, 1157594. <https://doi.org/10.3389/fpsyt.2023.1157594>

Meadows, A., & Higgs, S. (2019). Internalised weight stigma moderates the impact of a stigmatising prime on eating in the absence of hunger in higher- but not lower-weight individuals. *Frontiers in Psychology*, *10*, 1022. <https://doi.org/10.3389/fpsyg.2019.01022>

Meadows, A., & Higgs, S. (2019). The multifaceted nature of weight-related self-stigma: validation of the Two-Factor Weight Bias Internalization Scale (WBIS-2F). *Frontiers in Psychology*, *10*, 808. <https://doi.org/10.3389/fpsyg.2019.00808>

Meadows, A., & Higgs, S. (2020). A bifactor analysis of the Weight Bias Internalization Scale: What are we really measuring? *Body Image*, *33*, 137–151. <https://doi.org/10.1016/j.bodyim.2020.02.013>

Meadows, A., & Higgs, S. (2020). Internalized weight stigma and the progression of food addiction over time. *Body Image*, *34*, 67–71. <https://doi.org/10.1016/j.bodyim.2020.05.002>

Meadows, A., & Higgs, S. (2022). Challenging oppression: A social identity model of stigma resistance in higher-weight individuals. *Body Image*, *42*, 237–245. <https://doi.org/10.1016/j.bodyim.2022.06.004>

Meadows, A., Nolan, L. J., & Higgs, S. (2017). Self-perceived food addiction: Prevalence, predictors, and prognosis. *Appetite*, *114*, 282–298. <https://doi.org/10.1016/j.appet.2017.03.051>

Mehak, A., Friedman, A., & Cassin, S. E. (2018). Self-objectification, weight bias internalization, and binge eating in young women: Testing a mediational model. *Body Image*, *24*, 111–115. <https://doi.org/10.1016/j.bodyim.2018.01.002>

Mensinger, J., & Meadows, A. (2017). Internalized weight stigma mediates and moderates physical activity outcomes during a healthy living program for women with high body mass index. *Psychology of Sport and Exercise*, *30*, 64–72. <https://doi.org/10.1016/j.psychsport.2017.01.010>

Mensinger, J. L. (2021). Traumatic stress, body shame, and internalized weight stigma as mediators of change in disordered eating: A single-arm pilot study of the Body Trust® framework. *Eating Disorders*, *30*(6), 1–29. <https://doi.org/10.1080/10640266.2021.1985807>

Mensinger, J. L., Calogero, R. M., & Tylka, T. L. (2016). Internalized weight stigma moderates eating behavior outcomes in women with high BMI participating in a healthy living program. *Appetite*, *102*, 32–43. <https://doi.org/10.1016/j.appet.2016.01.033>

Mensinger, J. L., Shepherd, B. F., Schapiro, S., Aware, Y., Brochu, P. M., Calogero, R. M., & Tylka, T. L. (2023). Mediating effects of a weight-inclusive health promotion program on maladaptive eating in women with high body mass index. *Eating Behaviors*, *49*, 101730. <https://doi.org/10.1016/j.eatbeh.2023.101730>

Mensinger, J. L., Tylka, T. L., & Calamari, M. E. (2018). Mechanisms underlying weight status and healthcare avoidance in women: A study of weight stigma, body-related shame and guilt, and healthcare stress. *Body Image*, *25*, 139–147. <https://doi.org/10.1016/j.bodyim.2018.03.001>

Meskin, L., Colvin, M., & Hart, L. (2021). A pilot trial of confident body, confident child in the United States. *Families in Society*, *102*(2), 194–211. <https://doi.org/10.1177/1044389420947227>

Meyer, L. B., Waaddegaard, M., Lau, M. E., & Tjørnhøj-Thomsen, T. (2019). (Dis-)solving the weight problem in binge-eating disorder: systemic insights from three treatment contexts with weight stability, weight loss, and weight acceptance. *Qualitative Health Research*, *29*(4), 597–608. <https://doi.org/10.1177/1049732318764874>

Myre, M., Berry, T. R., Ball, G. D. C., & Hussey, B. (2020). Motivated, fit, and strong-using counter-stereotypical images to reduce weight stigma internalisation in women with obesity. *Applied Psychology: Health and Well-Being*, *12*(2), 335–356. <https://doi.org/10.1111/aphw.12187>

Myre, M., Glenn, N., & Berry, T. (2021). Exploring the impact of physical activity-related weight stigma among women with self-identified obesity. *Qualitative Research in Sport Exercise and Health*, *13*(4), 586–603. <https://doi.org/10.1080/2159676X.2020.1751690>

Nadhiroh, S., Nurmala, I., Pramukti, I., Tivany, S., Tyas, L., Zari, A., Poon, W., Siaw, Y., Kamolthip, R., Chirawat, P., & Lin, C. (2022). Weight stigma in Indonesian young adults: Validating the Indonesian versions of the Weight Self-Stigma Questionnaire and Perceived Weight Stigma Scale. *Asian Journal of Social Health and Behavior*, *5*(4), 169–179. <https://doi.org/10.4103/shb.shb_189_22>

Nadolsky K., Addison B., Agarwal M., Almandoz J. P., Bird M. D., DeGeeter Chaplin M., Garvey W. T., & Kyle T. K. (2023). American Association of Clinical Endocrinology consensus statement: Addressing stigma and bias in the diagnosis and management of patients with obesity/adiposity-based chronic disease and assessing bias and stigmatization as determinants of disease severity. *Endocrine Practice*, *29*(6), 417–427. <https://doi.org/10.1016/j.eprac.2023.03.272>

Nagpal, T. S., Ramos Salas, X., Vallis, M., Piccinini-Vallis, H., Adamo, K. B., Alberga, A. S., Bell, R. C., da Silva, D. F., Davenport, M. H., Gaudet, L., Incollingo Rodriguez, A. C., Liu, R. H., Myre, M., Nerenberg, K., Nutter, S., Russell-Mayhew, S., Souza, S. C. S., & Vilhan, C. (2021). Coming soon: An Internalized Weight Bias Assessment Scale for use during pregnancy. *Obesity*, *29*(5), 788–789. <https://doi.org/10.1002/oby.23169>

Nagpal, T. S., Salas, X. R., Vallis, M., Piccinini-Vallis, H., Alberga, A. S., Bell, R. C., Da Silva, D. F., Davenport, M. H., Gaudet, L., Rodriguez, A. C. I., Liu, R. H., Myre, M., Nerenberg, K., Nutter, S., Russell-Mayhew, S., Souza, S. C. S., Vilhan, C., & Adamo, K. B. (2022). Exploring weight bias internalization in pregnancy. *BMC Pregnancy and Childbirth*, *22*(1), 605. <https://doi.org/10.1186/s12884-022-04940-4>

Nakamura, Y., & Asano, M. (2023). Developing and validating a Japanese version of the Weight Self-Stigma Questionnaire. *Eating and Weight Disorders*, *28*(1), 44. <https://doi.org/10.1007/s40519-023-01573-0>

Nejati, B., Fan, C. W., Boone, W. J., Griffiths, M. D., Lin, C. Y., & Pakpour, A. H. (2021). Validating the Persian Intuitive Eating Scale-2 among breast cancer survivors who are overweight/obese. *Evaluation & the Health Professions*, *44*(4), 385–394. <https://doi.org/10.1177/0163278720965688>

Nicolau, J., Tofe, S., Bonet, A., Sanchis, P., Pujol, A., Ayala, L., Gil, A., & Masmiquel, L. (2023). Effects of weight stigma on BMI and inflammatory markers among people living with obesity. *Physiology & Behavior*, *262*, 114088. <https://doi.org/10.1016/j.physbeh.2023.114088>

Nightingale, B. A., & Cassin, S. E. (2023). Self-compassion may have benefits for body image among women with a higher body mass index and internalized weight bias. *Healthcare*, *11*(7). <https://doi.org/10.3390/healthcare11070970>

Nolan, L. J., & Eshleman, A. (2016). Paved with good intentions: Paradoxical eating responses to weight stigma. *Appetite*, *102*, 15–24. <https://doi.org/10.1016/j.appet.2016.01.027>

Noonan-Gunning, S. (2019). Social implications of weight bias internalisation: parents’ ultimate responsibility as consent, social division and resistance. *Frontiers in Psychology*, *10*, 2321. <https://doi.org/10.3389/fpsyg.2019.02321>

Nutter, S., Saunders, J., & Beharaj, G. (2023). Validating the Sociocultural Influences on Fear of Fat Scale (SI-FAT) on a racially and ethnically diverse sample of college women. *Body Image*, *47*, 101611. <https://doi.org/10.1016/j.bodyim.2023.08.003>

O’Brien, K. S., Latner, J. D., Puhl, R. M., Vartanian, L. R., Giles, C., Griva, K., & Carter, A. (2016). The relationship between weight stigma and eating behavior is explained by weight bias internalization and psychological distress. *Appetite*, *102*, 70–76. <https://doi.org/10.1016/j.appet.2016.02.032>

O’Hara, L., Ahmed, H., & Elashie, S. (2021). Evaluating the impact of a brief Health at Every Size (R)-informed health promotion activity on body positivity and internalized weight-based oppression. *Body Image*, *37*, 225–237. <https://doi.org/10.1016/j.bodyim.2021.02.006>

O’Hara, L., Alajaimi, B., & Alshowaikh, B. (2023). “I was bullied for being fat in every situation, in every outfit, at every celebration”: A qualitative exploratory study on experiences of weight-based oppression in Qatar. *Frontiers in Public Health*, *11*. <https://doi.org/10.3389/fpubh.2023.1015181>

Olson, K. L., Goldstein, S. P., Wing, R. R., Williams, D. M., Demos, K. E., & Unick, J. L. (2021). Internalized weight bias is associated with perceived exertion and affect during exercise in a sample with higher body weight. *Obesity Science and Practice*, *7*(4), 405–414. <https://doi.org/10.1002/osp4.494>

Olson, K. L., Landers, J. D., Thaxton, T. T., & Emery, C. F. (2019). The pain of weight-related stigma among women with overweight or obesity. *Stigma and Health*, *4*(3), 243–246. <https://doi.org/10.1037/sah0000137>

Olson, K. L., Lillis, J., Graham Thomas, J., & Wing, R. R. (2018). Prospective evaluation of internalized weight bias and weight change among successful weight-loss maintainers. *Obesity*, *26*(12), 1888–1892. <https://doi.org/10.1002/oby.22283>

Olson, K. L., & Mensinger, J. L. (2019). Weight-related stigma mediates the relationship between weight status and bodily pain: A conceptual model and call for further research. *Body Image*, *30*, 159–164. <https://doi.org/10.1016/j.bodyim.2019.07.005>

Olson, K. L., Panza, E., Lillis, J., & Wing, R. R. (2023). Association of weight-related stigmas with daily pain symptoms among individuals with obesity. *Annals of Behavioral Medicine*, *57*(3), 269–274. <https://doi.org/10.1093/abm/kaac025>

Olson, K. L., Thaxton, T. T., & Emery, C. F. (2018). Targeting body dissatisfaction among women with overweight or obesity: A proof-of-concept pilot study. *International Journal of Eating Disorders*, *51*(8), 973–977. <https://doi.org/10.1002/eat.22874>

Ozdemir, A. A., & Turkben, H. (2023). The relationship between weight self-stigma, depression and loneliness in people with obesity. *African Health Sciences*, *23*(3), 696–704. <https://doi.org/10.4314/ahs.v23i3.80>

Pakpour, A. H., Tsai, M. C., Lin, Y. C., Strong, C., Latner, J. D., Fung, X. C. C., Lin, C. Y., & Tsang, H. W. H. (2019). Psychometric properties and measurement invariance of the Weight Self-Stigma Questionnaire and Weight Bias Internalization Scale in children and adolescents. *International Journal of Clinical and Health Psychology*, *19*(2), 150–159. <https://doi.org/10.1016/j.ijchp.2019.03.001>

Palmeira, L., Cunha, M., & Pinto-Gouveia, J. (2018). The weight of weight self-stigma in unhealthy eating behaviours: The mediator role of weight-related experiential avoidance. *Eating and Weight Disorders*, *23*(6), 785–796. <https://doi.org/10.1007/s40519-018-0540-z>

Palmeira, L., Cunha, M., & Pinto-Gouveia, J. (2019). Processes of change in quality of life, weight self-stigma, body mass index and emotional eating after an acceptance-, mindfulness- and compassion-based group intervention (Kg-Free) for women with overweight and obesity. *Journal of Health Psychology*, *24*(8), 1056–1069. <https://doi.org/10.1177/1359105316686668>

Palmeira, L., Pinto-Gouveia, J., & Cunha, M. (2016). The role of weight self-stigma on the quality of life of women with overweight and obesity: A multi-group comparison between binge eaters and non-binge eaters. *Appetite*, *105*, 782–789. <https://doi.org/10.1016/j.appet.2016.07.015>

Palmeira, L., Pinto-Gouveia, J., & Cunha, M. (2017). Exploring the efficacy of an acceptance, mindfulness & compassionate-based group intervention for women struggling with their weight (Kg-Free): A randomized controlled trial. *Appetite*, *112*, 107–116. <https://doi.org/10.1016/j.appet.2017.01.027>

Palmeira, L., Pinto-Gouveia, J., Cunha, M., & Carvalho, S. (2017). Finding the link between internalized weight-stigma and binge eating behaviors in Portuguese adult women with overweight and obesity: The mediator role of self-criticism and self-reassurance. *Eating Behaviors, 26*, 50–54. <https://doi.org/10.1016/j.eatbeh.2017.01.006>

Panza, E., Fehling, K. B., Pantalone, D. W., Dodson, S., & Selby, E. A. (2021). Multiply marginalized: Linking minority stress due to sexual orientation, gender, and weight to dysregulated eating among sexual minority women of higher body weight. *Psychology of Sexual Orientation and Gender Diversity*, *8*(4), 420–428. <https://doi.org/10.1037/sgd0000431>

Panza, E., Lillis, J., Olson, K., van den Berg, J. J., Tashima, K., & Wing, R. R. (2022). HIV Status, Obesity, and Risk for Weight Stigma: Comparing Weight Stigma Experiences and Internalization Among Adults with Obesity with and Without HIV. *AIDS and Behavior*, *26*(3), 686–697. <https://doi.org/10.1007/s10461-021-03428-0>

Papadopoulos, S., & Brennan, L. (2015). Correlates of weight stigma in adults with overweight and obesity: A systematic literature review. *Obesity*, *23*(9), 1743–1760. <https://doi.org/10.1002/oby.21187>

Papadopoulos, S., Garcia, X., & Brennan, L. (2021). Evaluation of the psychometric properties of self-reported weight stigma measures: A systematic literature review. *Obesity Reviews*, *22*(8). <https://doi.org/10.1111/obr.13267>

Pape, M., Herpertz, S., Schroeder, S., Seiferth, C., Färber, T., Wolstein, J., & Steins-Loeber, S. (2021). Food addiction and its relationship to weight- and addiction-related psychological parameters in individuals with overweight and obesity. *Frontiers in Psychology*, *12*, 736454. <https://doi.org/10.3389/fpsyg.2021.736454>

Park, S., & Seo, K. (2023). Validity and reliability of the Korean version of the Weight Self-Stigma Questionnaire (WSSQ-K). *Nursing Reports*, *13*(2), 835–843. <https://doi.org/10.3390/nursrep13020073>

Parnarouskis, L., Jouppi, R. J., Cummings, J. R., & Gearhardt, A. N. (2021). A randomized study of effects of obesity framing on weight stigma. *Obesity*, *29*(10), 1625–1634. <https://doi.org/10.1002/oby.23247>

Pearl, R., Puhl, R., & Dovidio, J. (2017). Can legislation prohibiting weight discrimination improve psychological well-being? A preliminary investigation. *Analyses of Social Issues and Public Policy*, *17*(1), 84–104. <https://doi.org/10.1111/asap.12128>

Pearl, R. L., Bach, C., & Wadden, T. A. (2023). Development of a cognitive-behavioral intervention for internalized weight stigma. *Journal of Contemporary Psychotherapy*, *53*(2), 165–172. <https://doi.org/10.1007/s10879-022-09543-w>

Pearl, R. L., & Dovidio, J. F. (2015). Experiencing weight bias in an unjust world: Impact on exercise and internalization. *Health Psychology*, *34*(7), 741–749. <https://doi.org/10.1037/hea0000178>

Pearl, R. L., Hernandez, M., Bach, C., Groshon, L., & Wadden, T. A. (2023). Prevalence of diagnosed psychiatric disorders among adults who have experienced and internalized weight stigma. *Obesity Science and Practice*, *9*(6), 681–687. <https://doi.org/10.1002/osp4.700>

Pearl, R. L., Himmelstein, M. S., Puhl, R. M., Wadden, T. A, Wojtanowski, A. C., & Foster, G. D. (2019). Weight bias internalization in a commercial weight management sample: Prevalence and correlates. *Obesity Science and Practice*, *5*(4), 342–353. <https://doi.org/10.1002/osp4.354>

Pearl, R. L., Hopkins, C. H., Berkowitz, R. I., & Wadden, T. A. (2018). Group cognitive-behavioral treatment for internalized weight stigma: A pilot study. *Eating and Weight Disorders,* *23*(3), 357–362. <https://doi.org/10.1007/s40519-016-0336-y>

Pearl, R. L., & Lebowitz, M. S. (2014). Beyond personal responsibility: Effects of causal attributions for overweight and obesity on weight-related beliefs, stigma, and policy support. *Psychology & Health*, *29*(10), 1176–1191. <https://doi.org/10.1080/08870446.2014.916807>

Pearl, R. L., & Puhl, R. M. (2014). Measuring internalized weight attitudes across body weight categories: Validation of the modified weight bias internalization scale. *Body Image*, *11*(1), 89–92. <https://doi.org/10.1016/j.bodyim.2013.09.005>

Pearl, R. L., & Puhl, R. M. (2016). The distinct effects of internalizing weight bias: An experimental study. *Body Image*, *17*, 38–42. <https://doi.org/10.1016/j.bodyim.2016.02.002>

Pearl, R. L., & Puhl, R. M. (2018). Weight bias internalization and health: A systematic review. *Obesity Reviews*, *19*(8), 1141–1163. <https://doi.org/10.1111/obr.12701>

Pearl, R. L., Puhl, R. M., & Dovidio, J. F. (2015). Differential effects of weight bias experiences and internalization on exercise among women with overweight and obesity. *Journal of Health Psychology, 20*(12), 1626–1632. <https://doi.org/10.1177/1359105313520338>

Pearl, R. L., Puhl, R. M., Himmelstein, M. S., Pinto, A. M., & Foster, G. D. (2020). Weight stigma and weight-related health: associations of self-report measures among adults in weight management. *Annals of Behavioral Medicine*, *54*(11), 904–914. <https://doi.org/10.1093/abm/kaaa026>

Pearl, R. L., Puhl, R. M., Lessard, L. M., Himmelstein, M. S., & Foster, G. D. (2021). Prevalence and correlates of weight bias internalization in weight management: A multinational study. *SSM - Population Health*, *13*, 100755. <https://doi.org/10.1016/j.ssmph.2021.100755>

Pearl, R. L., & Wadden T. A. (2018). Weight stigma affects men too. *Obesity*, *26*(6), 949. <https://doi.org/10.1002/oby.22205>

Pearl, R. L., Wadden, T. A., Bach, C., Gruber, K., Leonard, S., Walsh, O. A., Tronieri, J. S., & Berkowitz, R. I. (2020). Effects of a cognitive-behavioral intervention targeting weight stigma: A randomized controlled trial. *Journal of Consulting and Clinical Psychology*, *88*(5), 470–480. <https://doi.org/10.1037/ccp0000480>

Pearl, R. L., Wadden, T. A., Bach, C., LaFata, E. M., Gautam, S., Leonard, S., Berkowitz, R. I., Latner, J. D., & Jakicic, J. M. (2023). Long-term effects of an internalized weight stigma intervention: A randomized controlled trial. *Journal of Consulting and Clinical Psychology*, *91*(7), 398–410. <https://doi.org/10.1037/ccp0000819>

Pearl, R. L., Wadden, T. A., Bach, C., Tronieri, J. S., & Berkowitz, R. I. (2020). Six-month follow-up from a randomized controlled trial of the Weight BIAS Program. *Obesity*, *28*(10), 1878–1888. <https://doi.org/10.1002/oby.22931>

Pearl, R. L., Wadden, T. A., Chao, A. M., Walsh, O., Alamuddin, N., Berkowitz, R. I., & Tronieri, J. S. (2019). Weight bias internalization and long-term weight loss in patients with obesity. *Annals of Behavioral Medicine*, *53*(8), 782–787. <https://doi.org/10.1093/abm/kay084>

Pearl, R. L., Wadden, T. A., Groshon, L. C., Fitterman-Harris, H. F., Bach, C., & LaFata, E. M. (2023). Refining the conceptualization and assessment of internalized weight stigma: A mixed methods approach. *Body Image*, *44*, 93–102. <https://doi.org/10.1016/j.bodyim.2022.12.002>

Pearl, R. L., Wadden, T. A., Hopkins, C. M., Shaw, J. A., Hayes, M. R., Bakizada, Z. M., Alfaris, N., Chao, A. M., Pinkasavage, E., Berkowitz, R. I., & Alamuddin, N. (2017). Association between weight bias internalization and metabolic syndrome among treatment-seeking individuals with obesity. *Obesity*, *25*(2), 317–322. <https://doi.org/10.1002/oby.21716>

Pearl, R. L., Wadden, T. A., & Jakicic, J. M. (2021). Is weight stigma associated with physical activity? A systematic review. *Obesity*, *29*(12), 1994–2012. <https://doi.org/10.1002/oby.23274>

Pearl, R. L., Wadden, T. A., Shaw Tronieri, J., Chao, A. M., Alamuddin, N., Bakizada, Z. M., Pinkasavage, E., & Berkowitz, R. I. (2018). Sociocultural and familial factors associated with weight bias internalization. *Obesity Facts*, *11*(2), 157–164. <https://doi.org/10.1159/000488534>

Pearl, R. L., White, M. A., & Grilo, C. M. (2014). Overvaluation of shape and weight as a mediator between self-esteem and weight bias internalization among patients with binge eating disorder. *Eating Behaviors*, *15*(2), 259–261. <https://doi.org/10.1016/j.eatbeh.2014.03.005>

Pearl, R. L., White, M. A., & Grilo, C. M. (2014). Weight bias internalization, depression, and self-reported health among overweight binge eating disorder patients. *Obesity*, *22*(5), E142-8. <https://doi.org/10.1002/oby.20617>

Petersen, J. M., Durward, C., & Levin, M. (2021). Weight-related psychological inflexibility as a mediator between weight self-stigma and health-related outcomes. *Bulletin of the Menninger Clinic*, *85*(3), 316–330. <https://doi.org/10.1521/bumc.2021.85.3.316>

Pico, M. L., Grunnet, L. G., Vinter, C. A., Aagaard-Hansen, J., & Kragelund Nielsen, K. (2023). Barriers and facilitators for sustainable weight loss in the pre-conception period among Danish women with overweight or obesity—A qualitative study. *BMC Public Health*, *23*(1), 1778. <https://doi.org/10.1186/s12889-023-16676-7>

Potts, S., Krafft, J., & Levin, M. E. (2022). A pilot randomized controlled trial of acceptance and commitment therapy guided self-help for overweight and obese adults high in weight self-stigma. *Behavior Modification*, *46*(1), 178–201. <https://doi.org/10.1177/0145445520975112>

Pötzsch, A., Rudolph, A., Schmidt, R., & Hilbert, A. (2018). Two sides of weight bias in adolescent binge-eating disorder: Adolescents’ perceptions and maternal attitudes. *International Journal of Eating Disorders*, *51*(12), 1339–1345. <https://doi.org/10.1002/eat.22982>

Prunty, A., Clark, M. K., Hahn, A., Edmonds, S., & O’Shea, A. (2020). Enacted weight stigma and weight self stigma prevalence among 3821 adults. *Obesity Research & Clinical Practice*, *14*(5), 421–427. <https://doi.org/10.1016/j.orcp.2020.09.003>

Prunty, A., Hahn, A., O’Shea, A., Edmonds, S., & Clark, M. K. (2023). Associations among enacted weight stigma, weight self-stigma, and multiple physical health outcomes, healthcare utilization, and selected health behaviors. *International Journal of Obesity*, *47*(1), 33–38. <https://doi.org/10.1038/s41366-022-01233-w>

Pudney, E. V., Himmelstein, M. S., & Puhl, R. M. (2019). The role of weight stigma in parental weight talk. *Pediatric Obesity*, *14*(10), e12534. <https://doi.org/10.1111/ijpo.12534>

Pudney, E. V., Himmelstein, M. S., Puhl, R. M., & Foster, G. D. (2020). Distressed or not distressed? A mixed methods examination of reactions to weight stigma and implications for emotional wellbeing and internalized weight bias. *Social Science & Medicine*, *249*, 112854. <https://doi.org/10.1016/j.socscimed.2020.112854>

Puhl, R. M., & Himmelstein, M. S. (2018). Adolescent preferences for weight terminology used by health care providers. *Pediatric Obesity*, *13*(9), 533–540. <https://doi.org/10.1111/ijpo.12275>

Puhl, R. M., & Himmelstein, M. S. (2018). Weight bias internalization among adolescents seeking weight loss: Implications for eating behaviors and parental communication. *Frontiers in Psychology*, *9*, 2271. <https://doi.org/10.3389/fpsyg.2018.02271>

Puhl, R. M., Himmelstein, M. S., Gorin, A. A., & Suh, Y. J. (2017). Missing the target: Including perspectives of women with overweight and obesity to inform stigma-reduction strategies. *Obesity Science and Practice*, *3*(1), 25–35. <https://doi.org/10.1002/osp4.101>

Puhl, R. M., Himmelstein, M. S., Hateley-Browne, J. L., & Speight, J. (2020). Weight stigma and diabetes stigma in U.S. adults with type 2 diabetes: Associations with diabetes self-care behaviors and perceptions of health care. *Diabetes Research and Clinical Practice*, *168*, 108387. <https://doi.org/10.1016/j.diabres.2020.108387>

Puhl, R. M., Himmelstein, M. S., Pearl, R. L., Wojtanowski, A. C., & Foster, G. D. (2019). Weight stigma among sexual minority adults: Findings from a matched sample of adults engaged in weight management. *Obesity*, *27*(11), 1906–1915. <https://doi.org/10.1002/oby.22633>

Puhl, R. M., Himmelstein, M. S., & Quinn, D. M. (2018). Internalizing weight stigma: Prevalence and sociodemographic considerations in US adults. *Obesity*, *26*(1), 167–175. <https://doi.org/10.1002/oby.22029>

Puhl, R. M., Himmelstein, M. S., & Speight, J. (2022). Weight stigma and diabetes stigma: Implications for weight-related health behaviors in adults with type 2 diabetes. *Clinical Diabetes*, *40*(1), 51–61. <https://doi.org/10.2337/cd20-0071>

Puhl, R. M., Lessard, L. M., Himmelstein, M. S., & Foster, G. D. (2021). The roles of experienced and internalized weight stigma in healthcare experiences: Perspectives of adults engaged in weight management across six countries. *PLoS One*, *16*(6), e0251566. <https://doi.org/10.1371/journal.pone.0251566>

Puhl, R. M., Lessard, L. M., Pearl, R. L., Grupski, A., & Foster, G. D. (2021). Policies to address weight discrimination and bullying: Perspectives of adults engaged in weight management from six nations. *Obesity*, *29*(11), 1787–1798. <https://doi.org/10.1002/oby.23275>

Puhl, R. M., Moss‐Racusin, C. A., & Schwartz, M. B. (2007). Internalization of weight bias: Implications for binge eating and emotional well‐being. *Obesity*, *15*(1), 19–23. <https://doi.org/10.1038/oby.2007.521>

Puhl, R. M., Moss-Racusin, C. A., Schwartz, M. B., & Brownell, K. D. (2007). Weight stigmatization and bias reduction: Perspectives of overweight and obese adults. *Health Education Research*, *23*(2), 347–358. <https://doi.org/10.1093/her/cym052>

Puhl, R. M., Quinn, D. M., Weisz, B. M., & Suh, Y. J. (2017). The role of stigma in weight loss maintenance among U.S. adults. *Annals Behavioral Medicine*, *51*(5), 754–763. <https://doi.org/10.1007/s12160-017-9898-9>

Puia, I., Stanculete, M., Hopulele-Petri, A., Muresan, D., & Puia, A. (2017). Patients’ perception of weight-related stigma in a Romanian sample. *Journal of Evidence-Based Psychotherapies*, *17*(2), 147–157. <https://doi.org/10.24193/jebp.2017.2.9>

Pullmer, R., Kerrigan, S. G., Grilo, C. M., & Lydecker, J. A. (2021). Factors linking perceived discrimination and weight bias internalization to body appreciation and eating pathology: A moderated mediation analysis of self-compassion and psychological distress. *Stigma and Health*, *6*(4), 494–501. <https://doi.org/10.1037/sah0000334>

Purton, T., Mond, J., Cicero, D., Wagner, A., Stefano, E., Rand-Giovannetti, D., & Latner, J. (2019). Body dissatisfaction, internalized weight bias and quality of life in young men and women. *Quality of Life Research*, *28*(7), 1825–1833. <https://doi.org/10.1007/s11136-019-02140-w>

Quinn, D. M., Puhl, R. M., & Reinka, M. A. (2020). Trying again (and again): Weight cycling and depressive symptoms in U.S. adults. *PLoS One*, *15*(9), e0239004. <https://doi.org/10.1371/journal.pone.0239004>

Ramirez, J. C., & Milan, S. (2016). Perceived size of friends and weight evaluation among low-income adolescents. *Journal of Behavioral Medicine*, *39*(2), 334–345. <https://doi.org/10.1007/s10865-015-9682-x>

Ramos Salas, X., Forhan, M., Caulfield, T., Sharma, A. M., & Raine, K. D. (2019). Addressing internalized weight bias and changing damaged social identities for people living with obesity. *Frontiers in Psychology*, *10*, 1409. <https://doi.org/10.3389/fpsyg.2019.01409>

Ratcliffe, D., & Ellison, N. (2015). Obesity and internalized weight stigma: A formulation model for an emerging psychological problem. *Behavioural and Cognitive Psychotherapy*, *43*(2), 239–252. <https://doi.org/10.1017/S1352465813000763>

Raves, D. M., Brewis, A., Trainer, S., Han, S. Y., & Wutich, A. (2016). Bariatric surgery patients’ perceptions of weight-related stigma in healthcare settings impair post-surgery dietary adherence. *Frontiers in Psychology*, *7*, 1497. <https://doi.org/10.3389/fpsyg.2016.01497>

Reinka, M. A., Quinn, D. M., & Puhl, R. M. (2021). Examining the relationship between weight controllability beliefs and eating behaviors: The role of internalized weight stigma and BMI. *Appetite*, *164*, 105257. <https://doi.org/10.1016/j.appet.2021.105257>

Remmert, J. E., Convertino, A. D., Roberts, S. R., Godfrey, K. M., & Butryn, M. L. (2019). Stigmatizing weight experiences in health care: Associations with BMI and eating behaviours. *Obesity Science and Practice*, *5*(6), 555–563. <https://doi.org/10.1002/osp4.379>

Roberto, C. A., Sysko, R., Bush, J., Pearl, R., Puhl, R. M., Schvey, N. A., & Dovidio, J. F. (2012). Clinical correlates of the weight bias internalization scale in a sample of obese adolescents seeking bariatric surgery. *Obesity*, *20*(3), 533–539. <https://doi.org/10.1038/oby.2011.123>

Robinson, E., Haynes, A., Sutin, A., & Daly, M. (2020). Self-perception of overweight and obesity: A review of mental and physical health outcomes. *Obesity Science and Practice*, *6*(5), 552–561. <https://doi.org/10.1002/osp4.424>

Rodgers, R. F. (2016). The role of the “Healthy Weight” discourse in body image and eating concerns: An extension of sociocultural theory. *Eating Behaviors*, *22*, 194–198. <https://doi.org/10.1016/j.eatbeh.2016.06.004>

Rojas-Sanchez, A., Sarrazin, P., Joet, G., Major, B., & Chalabaev, A. (2022). Motivational processes of the relationship between weight stigma and physical activity: A comparison between France and Mexico. *International Journal of Sport and Exercise Psychology*, *20*(4), 1117–1132. <https://doi.org/10.1080/1612197X.2021.1956565>

Romano, K. A., Heron, K. E., & Henson, J. M. (2021). Examining associations among weight stigma, weight bias internalization, body dissatisfaction, and eating disorder symptoms: Does weight status matter? *Body Image*, *37*, 38–49. <https://doi.org/10.1016/j.bodyim.2021.01.006>

Romano, K. A., Heron, K. E., Sandoval, C. M., Howard, L. M., MacIntyre, R. I., & Mason, T. B. (2022). A meta-analysis of associations between weight bias internalization and conceptually-related correlates: A step towards improving construct validity. *Clinical Psychology Review*, *92*, 102127. <https://doi.org/10.1016/j.cpr.2022.102127>

Romano, K. A., Heron, K. E., Sandoval, C. M., MacIntyre, R. I., Howard, L. M., Scott, M., & Mason, T. B. (2023). Weight bias internalization and psychosocial, physical, and behavioral health: A meta-analysis of cross-sectional and prospective associations. *Behavior Therapy*, 54(3), 539–556. <https://doi.org/10.1016/j.beth.2022.12.003>

Rosenbaum, D. L., & Bernstein, M. J. (2023). Evaluating the relationships between sexual orientation, weight-related teasing, weight bias internalization, and binge eating. *Psychology of Sexual Orientation and Gender Diversity*, *10*(4), 675–685. <https://doi.org/10.1037/sgd0000576>

Rossi, A. A., Manzoni, G. M., Pietrabissa, G., Di Pauli, D., Mannarini, S., & Castelnuovo, G. (2022). Weight stigma in patients with overweight and obesity: Validation of the Italian Weight Self-Stigma Questionnaire (WSSQ). *Eating and Weight Disorders*, *27*, 2459-2472. <https://doi.org/10.1007/s40519-022-01385-8>

Saffari, M., Chen, I.-H., Huang, P.-C., O’Brien, K. S., Hsieh, Y.-P., Chen, J.-K., Chang, Y.-H., Jiang, X., Bevan, N., Chang, Y.-L., Chen, J.-S., Tsai, C.-C., Griffiths, M. D., & Lin, C.-Y. (2023). Measurement invariance and psychometric evaluation of the tendency to avoid physical activity and sport scale (TAPAS) among Mainland Chinese university students. *Psychology Research and Behavior Management*, *16*, 3821–3836. <https://doi.org/10.2147/PRBM.S425804>

Saffari, M., Chen, J. S., Wu, H. C., Fung, X. C. C., Chang, C. C., Chang, Y. L., Kamolthip, R., Potenza, M. N., Lin, I. C., & Lin, C. Y. (2022). Effects of weight-related self-stigma and smartphone addiction on female university students’ physical activity levels. *International Journal of Environmental Research and Public Health*, *19*(5). <https://doi.org/10.3390/ijerph19052631>

Salvia, M. G., Ritholz, M. D., Craigen, K. L. E., & Quatromoni, P. A. (2023). Women’s perceptions of weight stigma and experiences of weight-neutral treatment for binge eating disorder: A qualitative study. *eClinicalMedicine*, *56*, 101811. <https://doi.org/10.1016/j.eclinm.2022.101811>

Sanchez, E., Ciudin, A., Sanchez, A., Gutierrez-Medina, S., Valdes, N., Flores, L., Mari-Sanchis, A., Goni, F., Sanchez, M., Nicolau, J., Munoz, C., Diaz-Trastoy, O., Cuatrecasas, G., Canizares, S., Comas, M., Lopez-Cano, C., & Lecube, A. (2023). Assessment of obesity stigma and discrimination among Spanish subjects with a wide weight range: The OBESTIGMA study. *Frontiers in Psychology*, *14*, 1209245. <https://doi.org/10.3389/fpsyg.2023.1209245>

Sarigiani, P., Olsavsky, A., Camarena, P., & Sullivan, S. (2020). Obesity and depressive symptoms in college women: Analysis of body image experiences and comparison to non-obese women. *International Journal of Adolescence and Youth*, *25*(1), 765–779. <https://doi.org/10.1080/02673843.2020.1740751>

Saunders, J. F., Nutter, S., & Russell-Mayhew, S. (2022). Examining the conceptual and measurement overlap of body dissatisfaction and internalized weight stigma in predominantly female samples: A meta-analysis and measurement refinement study. *Frontiers in Global Women’s Health*, *3*, 877554. <https://doi.org/10.3389/fgwh.2022.877554>

Schulte, E. M., Bach, C., Berkowitz, R. I., Latner, J. D., & Pearl, R. L. (2021). Adverse childhood experiences and weight stigma: Co-occurrence and associations with psychological well-being. *Stigma and Health*, *6*(4), 408–418. <https://doi.org/10.1037/sah0000341>

Schvey, N. A., Barmine, M., Bates, D., Oldham, K., Bakalar, J. L., Spieker, E., Maurer, D., Stice, E., Stephens, M., Tanofsky-Kraff, M., & Sbrocco, T. (2017). Weight stigma among active duty US military personnel with overweight and obesity. *Stigma and Health*, *2*(4), 281–291. <https://doi.org/10.1037/sah0000057>

Schvey, N. A., Sbrocco, T., Bakalar, J. L., Ress, R., Barmine, M., Gorlick, J., Pine, A., Stephens, M., & Tanofsky-Kraff, M. (2017). The experience of weight stigma among gym members with overweight and obesity. *Stigma and Health*, *2*(4), 292–306. <https://doi.org/10.1037/sah0000062>

Schvey, N. A., & White, M. A. (2015). The internalization of weight bias is associated with severe eating pathology among lean individuals. *Eating Behaviors*, *17*, 1–5. <https://doi.org/10.1016/j.eatbeh.2014.11.001>

Sedney, C., Cowher, A., Turiano, N. A., Cox, S., Dekeseredy, P., & Haggerty, T. (2023). Understanding the intersectional relationship of pain stigma, weight bias internalization, and clinical indicators in a rural population with back pain: A survey-based study. *World Neurosurgery*, *171*, e846-e851. <https://doi.org/10.1016/j.wneu.2022.12.125>

Selensky, J. C., & Carels, R. A. (2021). Weight stigma and media: An examination of the effect of advertising campaigns on weight bias, internalized weight bias, self-esteem, body image, and affect. *Body Image*, *36*, 95–106. <https://doi.org/10.1016/j.bodyim.2020.10.008>

Serap, O. A., & Bilge, A. (2023). Examining the effect of weight self-stigma on attitudes toward sexuality during pregnancy using structural equation modeling. *African Journal of Reproductive Health*, *27*(9), 87–95. <https://doi.org/10.29063/ajrh2023/v27i9.9>

Sevincer, G., Kaya, A., Bozkurt, S., Akin, E., & Kose, S. (2017). Reliability, validity, and factorial structure of the Turkish version of the Weight Self-Stigma Questionnaire (Turkish WSSQ). *Psychiatry and Clinical Psychopharmacology*, *27*(4), 386–392. <https://doi.org/10.1080/24750573.2017.1379717>

Shank, L. M., Schvey, N. A., Ekundayo, K., Schreiber-Gregory, D., Bates, D., Maurer, D., Spieker, E., Stephens, M., Tanofsky-Kraff, M., & Sbrocco, T. (2019). The relationship between weight stigma, weight bias internalization, and physical health in military personnel with or at high-risk of overweight/obesity. *Body Image*, *28*, 25–33. <https://doi.org/10.1016/j.bodyim.2018.11.003>

Sharp, M., Ward, L. G., Solar, C., Shea, C., Carels, R. A., & Dolbier, C. (2023). Internalized weight bias, weight-related experiences, and peripartum weight. *Journal of Midwifery and Women’s Health*, *68*(4), 490–498. <https://doi.org/10.1111/jmwh.13480>

Shonrock, A. T., Miller, J. C., Byrd, R., Sall, K. E., Jansen, E., Carraway, M., Campbell, L., & Carels, R. A. (2022). Experienced weight stigma, internalized weight bias, and maladaptive eating patterns among heterosexual and sexual minority individuals. *Eating and Weight Disorders*, *27*(8), 3487–3497. <https://doi.org/10.1007/s40519-022-01486-4>

Sienko, R. M., Saules, K. K., & Carr, M. M. (2016). Internalized weight bias mediates the relationship between depressive symptoms and disordered eating behavior among women who think they are overweight. *Eating Behaviors*, *22*, 141–144. <https://doi.org/10.1016/j.eatbeh.2016.06.002>

Sonneville, K. R., Rose, K. L., Lambrecht, N. J., Barry, M. R., Weeks, H. M., & Leung, C. W. (2021). Weight bias among public health trainees. *Public Health Nutrition*, *24*(6), 1566–1569. <https://doi.org/10.1017/S1368980020004097>

Soulliard, Z. A., Brode, C., Tabone, L. E., Abunnaja, S., Szoka, N., & Cox, S. (2021). The role of body appreciation, weight bias internalization, and disordered eating behaviors among presurgical bariatric patients. *Surgery for Obesity and Related Diseases*, *17*(5), 1000–1007. <https://doi.org/10.1016/j.soard.2020.12.012>

Soulliard, Z. A., Brode, C., Tabone, L. E., Szoka, N., Abunnaja, S., & Cox, S. (2021). Disinhibition and subjective hunger as mediators between weight bias internalization and binge eating among pre-surgical bariatric patients. *Obesity Surgery*, *31*(2), 797–804. <https://doi.org/10.1007/s11695-020-05023-z>

Stewart, S. F., & Ogden, J. (2021). The role of social exposure in predicting weight bias and weight bias internalisation: An international study. *International Journal of Obesity*, *45*(6), 1259–1270. <https://doi.org/10.1038/s41366-021-00791-9>

Stewart, S. F., & Ogden, J. (2021). What are weight bias measures measuring? An evaluation of core measures of weight bias and weight bias internalisation. *Health Psychology Open*, *8*(2), 20551029211029149. <https://doi.org/10.1177/20551029211029149>

Sturgess, C. M. B., & Stinson, D. A. (2022). Fat embodiment for resistance and healing from weight stigma. *Body Image*, *41*, 52–57. <https://doi.org/10.1016/j.bodyim.2022.02.007>

Sun, M., Peng, J., & Lommel, L. (2022). Prevalence and correlates of weight stigma among postpartum women in China. *International Journal of Environmental Research and Public Health*, *19*(22), 14692. <https://doi.org/10.3390/ijerph192214692>

Sutin, A., & Terracciano, A. (2015). Body weight misperception in adolescence and incident obesity in young adulthood. *Psychological Science*, *26*(4), 507–511. <https://doi.org/10.1177/0956797614566319>

Täuber, S., Gausel, N., & Flint, S. W. (2018). Weight bias internalization: The maladaptive effects of moral condemnation on intrinsic motivation. *Frontiers in Psychology*, *9*, 1836. <https://doi.org/10.3389/fpsyg.2018.01836>

Vartanian, L. R., Pinkus, R. T., & Smyth, J. M. (2018). Experiences of weight stigma in everyday life: Implications for health motivation. *Stigma and Health*, *3*(2), 85–92. <https://doi.org/10.1037/sah0000077>

Verhaak, A. M. S., Ferrand, J., Puhl, R. M., Tishler, D. S., Papasavas, P. K., & Umashanker, D. (2022). Experienced weight stigma, internalized weight bias, and clinical attrition in a medical weight loss patient sample. *International Journal of Obesity*, *46*, 1241–1243. <https://doi.org/10.1038/s41366-022-01087-2>

Wagner, A. F., Butt, M., & Rigby, A. (2020). Internalized weight bias in patients presenting for bariatric surgery. *Eating Behaviors*, *39*, 101429. <https://doi.org/10.1016/j.eatbeh.2020.101429>

Walsh, O. A., Wadden, T. A., Tronieri, J. S., Chao, A. M., & Pearl, R. L. (2018). Weight bias internalization is negatively associated with weight-related quality of life in persons seeking weight loss. *Frontiers in Psychology*, *9*, 2576. <https://doi.org/10.3389/fpsyg.2018.02576>

Wang, S. B., Lydecker, J. A., & Grilo, C. M. (2017). Rumination in patients with binge-eating disorder and obesity: Associations with eating-disorder psychopathology and weight-bias internalization. *European Eating Disorders Review*, *25*(2), 98–103. <https://doi.org/10.1002/erv.2499>

Warnick, J. L., Darling, K. E., West, C. E., Jones, L., & Jelalian, E. (2022). Weight stigma and mental health in youth: A systematic review and meta-analysis. *Journal of Pediatric Psychology*, *47*(3), 237–255. <https://doi.org/10.1093/jpepsy/jsab110>

Watson, C., Riazi, A., & Ratcliffe, D. (2020). Exploring the experiences of women who develop restrictive eating behaviours after bariatric surgery. *Obesity Surgery*, *30*(6), 2131–2139. <https://doi.org/10.1007/s11695-020-04424-4>

Weaver, L. J., & Trainer, S. (2017). Shame, blame, and status incongruity: Health and stigma in rural Brazil and the urban United Arab Emirates. *Culture, Medicine, and Psychiatry*, *41*(3), 319–340. <https://doi.org/10.1007/s11013-016-9518-3>

Webb, J. B., Padro, M. P., Thomas, E. V., Davies, A. E., Etzel, L., Rogers, C. B., & Heredia, N. I. (2022). Yoga at every size: A preliminary evaluation of a brief online size-inclusive yoga and body gratitude journaling intervention to enhance positive embodiment in higher weight college women. *Frontiers in Global Women’s Health*, *3*, 852854. <https://doi.org/10.3389/fgwh.2022.852854>

Webb, J. B., & Hardin, A. S. (2016). An integrative affect regulation process model of internalized weight bias and intuitive eating in college women. *Appetite*, *102*, 60–69. <https://doi.org/10.1016/j.appet.2016.02.024>

Weineland, S., Lillis, J., & Dahl, J. (2013). Measuring experiential avoidance in a bariatric surgery population—Psychometric properties of AAQ-W. Obes Res Clin Pract, 7(6), e464-75. <https://doi.org/10.1016/j.orcp.2012.06.002>

Welzel, F. D., Bär, J., Stein, J., Löbner, M., Pabst, A., Luppa, M., Grochtdreis, T., Kersting, A., Blüher, M., Luck-Sikorski, C., König, H. H., & Riedel-Heller, S. G. (2021). Using a brief web-based 5A intervention to improve weight management in primary care: Results of a cluster-randomized controlled trial. *BMC Family Practice*, *22*(1), 61. <https://doi.org/10.1186/s12875-021-01404-0>

Wetzel, K. E., & Himmelstein, M. S. (2023). Constant vigilance: The impact of weight stigma, vigilance, and internalization on maladaptive eating behaviors. *Health Psychology*, *42*(10), 712–722. <https://doi.org/10.1037/hea0001324>

Williams, O., & Annandale, E. (2019). Weight bias internalization as an embodied process: understanding how obesity stigma gets under the skin. *Frontiers in Psychology*, *10*, 953. <https://doi.org/10.3389/fpsyg.2019.00953>

Winter, V. R., Hood, A., Sorensen, B. L., & Trout, K. E. (2023). Sexual and reproductive health cancer screening avoidance: The role of body image. *Body Image*, *45*, 362–368. <https://doi.org/10.1016/j.bodyim.2023.04.001>

Wong, P., Hsieh, Y., Ng, H., Kong, S., Chan, K., Au, T., Lin, C., & Fung, X. (2019). Investigating the self-stigma and quality of life for overweight/obese children in Hong Kong: A preliminary study. *Child Indicators Research*, *12*(3), 1065–1082. <https://doi.org/10.1007/s12187-018-9573-0>

Xu, P., Chen, J. S., Chang, Y. L., Wang, X., Jiang, X., Griffiths, M. D., Pakpour, A. H., & Lin, C. Y. (2022). Gender differences in the associations between physical activity, smartphone use, and weight stigma. *Frontiers in Public Health*, *10*, 862829. <https://doi.org/10.3389/fpubh.2022.862829>

Zagaria, A., Cerolini, S., Mocini, E., & Lombardo, C. (2023). The relationship between internalized weight stigma and physical and mental health-related quality of life in a large sample of women: A structural equation modeling analysis. *Eating and Weight Disorders*, *28*(1), 52. <https://doi.org/10.1007/s40519-023-01582-z>

Zagaria, A., Mocini, E., Cerolini, S., Donini, L. M., Castelnuovo, G., Manzoni, G. M., Pietrabissa, G., & Lombardo, C. (2022). A validation study of the Italian version of the Attitudes Toward Obese Persons (I-ATOP) questionnaire. *Obesity Research and Clinical Practice*, *16*(3), 262–268. <https://doi.org/10.1016/j.orcp.2022.05.004>

Zhang, B. G., & Qian, X. F. (2022). Weight self-stigma and engagement among obese students in a physical education class. *Frontiers in Psychology*, *13*, 1035827. <https://doi.org/10.3389/fpsyg.2022.1035827>

Zhu, X., Smith, R. A., & Buteau, E. (2022). A meta-analysis of weight stigma and health behaviors. *Stigma and Health*, *7*(1), 1–13. <https://doi.org/10.1037/sah0000352>

Zuba, A., & Warschburger, P. (2017). The role of weight teasing and weight bias internalization in psychological functioning: A prospective study among school-aged children. *European Child & Adolescent Psychiatry*, *26*(10), 1245–1255. <https://doi.org/10.1007/s00787-017-0982-2>

Zuba, A., & Warschburger, P. (2018). Weight bias internalization across weight categories among school-aged children: Validation of the Weight Bias Internalization Scale for children. *Body Image*, *25*, 56–65. <https://doi.org/10.1016/j.bodyim.2018.02.008>
